# Selection index theory for populations under directional and stabilizing selection

**DOI:** 10.1186/s12711-023-00776-4

**Published:** 2023-02-03

**Authors:** Robin Wellmann

**Affiliations:** Department of Animal Genetics and Breeding, University of Hohenheim, Stuttgart, Germany

## Abstract

**Background:**

The purpose of a selection index is that its use to select animals for breeding maximizes the profit of a breed in future generations. The profit of a breed is in general a quantity that predicts the satisfaction of future owners with their breed, and the satisfaction of the consumers with the products that are produced by the breed. Many traits, such as conformation traits and product quality traits have intermediate optima. Traditional selection index theory applies only to directional selection and cannot achieve any further improvement once the trait means have reached their optima. A well-founded theory is needed that extends the established selection index theory to cover directional as well as stabilizing selection as limiting cases, and that can be applied to maximize the profit of a breed in both situations.

**Results:**

The optimum selection index shifts the trait means towards the optima and, in the case of stabilizing selection, decreases the phenotypic variance, which causes the phenotypes to be closer to the optimum. The optimum index depends not only on the breeding values, but also on the squared breeding values, the allele contents of major quantitative trait loci (QTL), the QTL heterozygosities, the inbreeding coefficient of the animal, and the kinship of the animal with the population.

**Conclusion:**

The optimum selection index drives the alleles of major QTL to fixation when the trait mean approaches the optimum because decreasing the phenotypic variance shifts the trait values closer to the optimum, which increases the profit of the breed. The index weight on the kinship coefficient balances the increased genetic gain that can be achieved in future generations by outcrossing, and the increased genetic gain that can be achieved under stabilizing selection by reducing the phenotypic variance. In a model with dominance variance, it can also account for the effect of inbreeding depression. The combining ability between potential mating partners, which predicts the total merit of their offspring, could become an important parameter for mate allocation that could be used to further shift the phenotypes towards their optimum values.

**Supplementary Information:**

The online version contains supplementary material available at 10.1186/s12711-023-00776-4.

## Background

The objective of breeding programs of domestic animals is to increase the suitability of a breed for its intended future use, which is measured by its total merit. The total merit of an animal is calculated by a total merit function $\text{TM}({\bf{y}}_{i})$, which in general is a non-linear function of the animal’s phenotype ${\bf{y}}_{i}$ [1]. As different breeds are kept for different purposes, each breed has its own total merit function. Breeding programs aim at increasing the average total merit of the animals, which is also called the breed’s profit. The breed’s profit is obtained by integrating the total merit function over the probability distribution of the traits [2, 3]. The resulting profit function $\phi$ can depend on various parameters, including the vector ${\varvec\upmu }$ of the trait means.

The breeding program of a breed defines a policy that is followed for selecting animals for breeding. A commonly used selection method is index selection. Index selection relies on a selection index which, in general, is a function of an animal’s estimated genetic values. It predicts the effect of using that animal for breeding on the breed’s profit when mating is at random [4]. This selection method is based on calculating a selection index for each animal and selecting animals for breeding whose selection indices exceed a certain threshold value. The breed’s profit changes with time as the breeding program proceeds, and software has been developed to predict selection responses [5]. A major task of many breeding programs is the design of a selection index that maximizes the genetic gain in the breed’s profit.

The technical terms used in this context are sometimes confusing. For example, the profit function and the total merit function do not necessarily measure profit or merit in monetary terms, but rather predict the suitability of a breed or animal for people who are expected to be typical future owners of the breed. In addition, the selection index is referred to by some authors as the total merit index. We do not use that terminology here because the reader could easily confuse an animal’s total merit with its total merit index.

Selection index theory was founded by Smith [6] and by Hazel [7] and his supervisor Lush. It is the standard tool used in livestock breeding to combine information on different traits into a single value. The profit function is traditionally approximated by a linear function in the vicinity of the vector ${\varvec\upmu }_0$ with current trait means, i.e., $\phi ({\varvec\upmu })\approx \phi ({{\varvec\upmu }}_0) + {\bf{v}}({{\varvec\upmu }}_0)^\top ({\varvec\upmu }-{{\varvec\upmu }}_0)$. The aggregate genotype of an animal is then defined as $T_i={\bf{v}}({{\varvec\upmu }}_0)^\top {\bf{TBV}}_i$, where ${\bf{TBV}}_i$ is the *K*-vector with true breeding values for the traits included in vector ${\varvec\upmu }$, and ${\bf{v}}({{\varvec\upmu }}_0)$ is called the vector with economic weights. The rationale behind this approach is that the aggregate genotype should measure the effect, using the animal has on the profit of the breed in the next generation. It can be seen as follows that the aggregate genotype serves indeed this purpose. The vector with trait means in the next generation is ${{\varvec\upmu }}_1(\bf{c})={{\upmu }}_0 + \bf{TBV}^\top {\bf{c}}$, where **c** is the *N*-vector with genetic contributions of the selection candidates, and $\bf{TBV}$ is the $N\times K$-matrix with their true breeding values, which are defined here to have a mean of zero. Thus, the genetic contribution $c_i$ of an animal equals the proportion of haplotypes in the next generation that originate from that animal. The aggregate genotype should measure the increase of the breed’s profit that results from increasing the genetic contribution $c_i$ of the animal beyond its default contribution $\tilde{c}_i$, so $T_i$ could be defined as the partial derivative $\frac{\partial }{\partial c_i} \phi ({{\varvec\upmu }}_1(\bf{c}))$ evaluated at $\tilde{\bf{c}}$. Application of the chain rule shows that indeed:

$$\begin{aligned} T_i=\frac{\partial \phi }{\partial {\varvec\upmu }_1} (\tilde{{\varvec\upmu }}_1) \frac{\partial {\varvec\upmu }_1}{\partial c_i} (\tilde{\bf{c}})= {\bf{v}}(\tilde{{\varvec\upmu }}_1)^\top {\bf{TBV}}_i= {\bf{v}}({{\varvec\upmu }}_0)^\top {\bf{TBV}}_i, \end{aligned}$$

provided that the vector $\tilde{\bf{c}}$ with default contributions is chosen such that $\tilde{{\varvec\upmu }}_1={{\varvec\upmu }}_0$. The selection index $I_i={\bf{b}}_i^\top {\bf{EBV}}_i$ of animal *i* is then obtained as the linear function of the estimated breeding values that has the highest correlation with the currently used aggregate genotype. The correlation is maximized for vector ${\bf{b}}_i={\bf{P}}_{i}^{-1}{\bf{G}}_i {\bf{v}}{({{\varvec\upmu} }}_0)$, where ${\bf{G}}_i$ is the matrix with covariances between EBV and TBV, and ${\bf{P}}_i$ is the covariance matrix of the EBV for the class of animals to which animal *i* belongs [8, 9]. Thus, the optimum vector ${{\bf{b}}_i}$ with weights of the traits in the selection index can depend on animal *i*, while the vector ${\bf{v}}({{\varvec\upmu }}_0)$ with economic weights does not depend on the animal. The use of this selection index maximizes short-term genetic progress towards the stated goal [10]. In practice, the permitted weights of the traits are often restricted to ensure that breeding progress of all traits is in the desired direction [11].

However, sometimes breeding programs do not focus on maximizing the short-term breeding progress, but on maximizing the breeding progress that can be achieved until the end of the planning horizon of the breeding program. This problem was solved by Moav and Hill [12] for the special case of two traits. It was later extended to any number of traits, and search algorithms for finding the optimum index weights were derived [13, 14]. The method of Moav and Hill [12], however, is inflexible because the genetic gain at the end of the planning horizon of the breeding program is maximized, while in practice, genetic gains in future generations are discounted. This leads to more emphasis being placed on the short-term than on the long-term response [4]. The generalized optimization problem with discounted future genetic gains was solved by [15] under the simplifying assumption that genetic variances and covariances do not change over time. In this case, the optimum index weights change in each generation.

The choice of the method for deriving the selection index could become critical when the profit function is highly non-linear, which is the case for traits under stabilizing selection. Various traits, such as meat quality traits (drip loss, color rating, intramuscular fat content, and pH) in pigs, egg weight and egg shell strength in layer chickens, and milking speed in dairy cattle, are subject to stabilizing selection. Stabilizing selection is also common for conformation traits. In addition, various performance traits, such as litter size in pigs and milk yield in dairy cows, may approach their optima in the foreseeable future and thus experience a transition from directional to stabilizing selection.

All above-mentioned authors implicitly assumed that the genetic parameters do not change over time. However, as stabilizing selection could be facilitated by inbreeding, it is clear that this assumption is wrong. Nevertheless, this assumption has led practitioners to believe that the vector with breeding values is a sufficient statistic from which the optimum selection index can be calculated. In this paper, we show that the true breeding values are not a sufficient statistic because a breeding animal changes the phenotypic mean and variance of a trait in future generations not only via its breeding values.

While the selection index can be used for determining whether an animal should be used for breeding, and how often it should be used, mate allocation can be based on a different parameter, which we call the combining ability between the sire and the dam. The combining ability of two potential mating partners is the expected total merit of their offspring, which can be decomposed into their general and specific combining abilities [16]. Mate selection based on combining abilities improves the closeness of the phenotypes to their optima.

The aim of this paper is to generalize selection index theory to a situation that assumes a realistic response of the genetic variances of the traits to selection decisions. For clarity, only the simplest models are considered that can provide interesting results, including a purely additive model with a polygenic term and contributions from large, known quantitative trait loci (QTL). The developed selection index theory is applicable to both directional and stabilizing selection. The structure of the paper is as follows. First, the general theory is introduced and the optimization problem is defined as finding the optimum breeding policy that maximizes the expected profit of the breed in future generations. Second, the general theory is applied to the special case of a distance-based total merit function. Third, the information on the selection candidates on which the optimum selection index could depend is determined for different underlying genetic models. Finally, the optimum selection index is estimated for a simulated population and compared with the conventional selection index. Symbols that are frequently used in this paper are summarized in Table 1. Proofs of the formulas are in Additional file 1. Although the optimum selection index is characterized under the assumption of random mating, the paper also provides formulas needed for optimizing mate allocation. Note that, in the following, we adopt a more general notation and write $\phi (\varvec\upxi )$ instead of $\phi ({{\varvec\upmu }})$, where $\varvec{\upxi }$ is the parameter of the phenotypic distribution that typically contains ${{\varvec\upmu }}$.

**Table 1:** List of frequently used symbols

Symbols	Meaning
Counts	
*O*	Number of breeding objectives
*K*	Number of traits
*Q*	Number of known biallelic QTL
*N*	Number of selection candidates
Information on selection candidates	
${\bf{y}}_{i}$	*K*-vector with trait values of animal *i*
${\bf{TBV}}_i$	*K*-vector with true breeding values of animal *i*
${\bf{EBV}}_i$	*K*-vector with estimated breeding values of animal *i*
${\text{MV}}_{ik}$	True Mendelian sampling variance of the haplotypes transmitted by animal *i* for trait *k*
$F_i$	Inbreeding coefficient of animal *i*
$\overline{f}_i$	Average kinship of animal *i* with animals of the opposite sex
$X_{iq}$	Allele content of animal *i* for QTL *q*
$H_{iq}$	Indicator for heterozygosity of animal *i* at QTL *q*
Parameters related to the total merit	
$\text{TM}({\bf{y}}_{i})$	Total merit of animal *i* with phenotype vector ${\bf{y}}_{i}$
$\phi (\varvec{\upxi })$	Profit of a breed whose phenotypic distribution is defined by $\varvec{\upxi }$
$\phi _{n}(\varvec{\uppi})$	Expected profit of the breed after *n* generations of selection under breeding policy $\varvec{\uppi}$
${\bf{Opt}}_o$	*K*-vector with optimum trait values for breeding objective *o*
$\omega _{ok}$	Weight of trait *k* for breeding objective *o*
Parameters related to index selection	
$\varvec{\uppi}$	Breeding policy that provides the selection index for a given state ${\varvec{{\uptheta}}}$ of the breeding program
$\bf{c}$	Vector with genetic contributions of the selection candidates.
$f(\varvec{\uppi})=\tilde{f}_{\varvec{\uppi}}(\bf{c}_{\varvec{\uppi}})$	Objective function. The function weights the expected profit in future generations.
$T_i$	Aggregate genotype of animal *i*
$I_{ i}$	Selection index of animal *i*
$T_{ij}$	True combining ability of male *i* with female *j*
$C_{ij}$	Estimated combining ability of male *i* with female *j*
Parameters for characterizing the population	
${\varvec{{\uptheta}}_n^{\varvec{\uppi}}}$	State of the population in generation *n*
${\varvec{\upxi }}_n^{\varvec{\uppi}}$	Parameter of the phenotypic distribution in generation *n*
$\mu _{nk}$	Expected mean of trait *k* in generation *n*
$\sigma _{\text{P}nk}^2$	Expected phenotypic variance of trait *k* in generation *n*

## Theory

### The setting

The state $\varvec{\uptheta}\in \Theta$ of a population is an *L*-vector that characterizes the state of the breeding program such that it provides the information needed for computing the vector ${\varvec\upmu }(\varvec\uptheta)\in \mathbb {R}^K$ with trait means, the vector ${\varvec\upsigma }_{\text{P}}^2(\varvec\uptheta)\in \mathbb {R}^K$ with phenotypic variances, and the vector ${\varvec\upsigma }_{\text{G}}^2(\varvec\uptheta)\in \mathbb {R}^K$ with genetic variances. Depending on the underlying genetic model, ${\varvec\uptheta}$ might provide additional population parameters, such as QTL allele frequencies, QTL heterozygosities, the average kinship, and the average inbreeding of the population. The phenotypic and genetic correlations could also be included in vector ${\varvec\uptheta}$ if they are not assumed to be constant.

Each animal *i* has an estimated genetic value $\hat{\bf{u}}_i$ that predicts its unknown true genetic value ${\bf{u}}_i$. The vector $\hat{\bf{u}}_i$ includes the estimated breeding values of the selection candidate, while ${\bf{u}}_i$ includes the true breeding values. However, depending on the underlying genetic model, the vectors ${\bf{u}}_i$ and $\hat{\bf{u}}_i$ may include additional information on the selection candidate such as the allele contents of large QTL. Vector ${\bf{u}}_i$ is in general a vector of information sources that includes all information on the animal that can affect its aggregate genotype, and $\hat{\bf{u}}_i$ is an estimation thereof. The vectors ${\bf{u}}_i,\hat{\bf{u}}_i$, and the population’s current state ${\varvec\uptheta}_0$ are removed in some of the following definitions to keep them readable.

The objective of a breeding program is assumed to maximize the breed’s profit in future generations. This requires determining the most appropriate breeding policy $\dot{\varvec\uppi}$ from a set $\Pi$ of permissible breeding policies, which is then used to move the population from its current state ${\varvec\uptheta}_0\in \Theta$ into the desired final state $\breve{\varvec\uptheta}\in \Theta$. A breeding policy $\varvec\uppi\in \Pi$ takes the state $\varvec\uptheta$ of the population into account, and determines which animals should be selected for breeding based on the genetic information $\hat{\bf{u}}_i$ that is available on each selection candidate *i*, and how they should be mated to produce the next generation. This paper considers a random mating population with truncation selection based on a selection index, so each breeding policy $\varvec\uppi=(T_{\varvec\uptheta},\text{tr}_{\varvec\uptheta})_{{\varvec\uptheta}\in \Theta}$ provides for a given state $\varvec\uptheta$ of the population a point of truncation $\text{tr}_{\varvec\uptheta}$ and an aggregate genotype function $T_{\varvec\uptheta}$. This function provides the aggregate genotype $T_i=T_{\varvec\uptheta}({\bf{u}}_i)$ of each animal *i*. It is used to calculate the selection index $I_i={\bf{b}}_i^\top \hat{\bf{u}}_i$ of the animal that has the highest correlation with its aggregate genotype. Animal *i* will be selected for breeding, if $I_i>\text{tr}_{\varvec\uptheta}$.

The selection candidates affect the population parameters in future generations via the genetic contributions they make to the next generation. Each contribution vector $\bf{c}$ has a representation $\bf{c}={\bf{c}}_\text{m}+{\bf{c}}_\text{f}$, where the vector ${\bf{c}}_\text{m}$ with male contributions equals zero for females, and the vector ${\bf{c}}_\text{f}$ with female contributions equals zero for males. In order to simplify notations, we denote with ${\text{s}}_i\in \{\text{m},\text{f}\}$ the sex of animal *i* and with $\overline{\text{s}}_i$ the opposite sex. Thus, we can write $\bf{c}={\bf{c}}_{\text{s}_i}+\bf{c}_{\overline{\text{s}}_i}$.

### The optimization problem

Let $\text{TM}(\bf{y})$ denote the total merit of an animal with phenotype ${\bf{y}}\in {\mathbb {R}}^K$, which predicts the satisfaction that typical future owners of the breed have with that animal. The expected profit $\phi _{n}(\varvec{\uppi})$ of the breed after $n\ge 1$ generations of selection by following breeding policy $\varvec{\uppi}$ equals the expected total merit of an animal that is randomly chosen from that generation. That is,

$$\begin{aligned} \phi _{n}(\varvec\uppi)= & {} E_{\boldsymbol{\uppi}}(\text{TM}({\bf{Y}}_{n})|\varvec\uptheta_0,{\bf{u}},\hat{\bf{u}}), \end{aligned}$$

where ${\bf{Y}}_{n}$ is the *K*-vector with phenotypic values of an animal that is randomly chosen from generation *n*. The current generation is $n=0$. Conditioning on the estimated genetic values $\hat{\bf{u}}=(\hat{\bf{u}}_1,\hat{\bf{u}}_2,...)$ is needed because selection decisions depend on the estimated genetic values of the selection candidates, while conditioning on the unknown true genetic values ${\bf{u}}=({\bf{u}}_1,{\bf{u}}_2,...)$ is needed because the state of the population in the next generation depends on the true genetic values of the selection candidates. The problem faced by animal breeders is to find the optimum breeding policy $\dot{\varvec\uppi}\in \Pi$ that maximizes the objective function:

$$\begin{aligned} f(\varvec\uppi)= & {} \sum \limits _{n=1}^\infty \zeta _n \phi _{n}(\varvec\uppi), \end{aligned}$$

where $\zeta _n$ is the importance placed on the profit of the breed in generation *n* [15]. The sequence $\varvec\zeta=(\zeta _n)_{n\ge 1}$, which satisfies $\zeta _n\ge 0$ and $\sum _n \zeta _n=1$, is needed for balancing short-term genetic gain vs. long-term genetic gain. For example, if the profit after $n'$ generations of selection should be maximized, then $\boldsymbol{\zeta }$ is chosen as the unit vector with $\zeta _{n'}=1$.

### The objective function

**Fig. 1:**
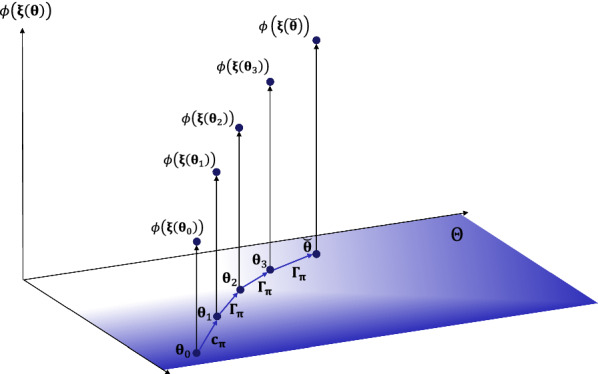
Illustrative example. The high-dimensional state space $\Theta$ is symbolized by the horizontal plane. The state ${\varvec\uptheta}_n\in \Theta$ of the population changes from one generation to the next in accordance with the breeding policy ${\varvec\uppi}$ until the desired final state $\breve{\varvec\uptheta}$ is reached. The breed’s profit $\phi (\varvec\upxi ({\varvec\uptheta}_n))$ in each generation is shown on the vertical axis. While the state ${\varvec\uptheta}_1$ in the first generation is determined by the vector $\bf{c}_{\varvec\uppi}$ with genetic contributions of the selection candidates, the change in state from one generation to the next in the following generations is given by a mapping $\Gamma _{\varvec\uppi}:\Theta \rightarrow \Theta$

The computation of the objective function requires prediction of the breed’s profit $\phi _{n}(\varvec\uppi)$ after *n* generations of selection under a given breeding policy $\varvec\uppi$. This section provides a general formula for computation. In large populations, the state of the population can be assumed to change deterministically. Then, the parameter ${\varvec\upxi }_n^{\varvec\uppi}\in \Xi$ of the phenotypic distribution after *n* generations of selection under breeding policy $\varvec\uppi$ is a non-random parameter, and the objective function has the alternative representation:

$$\begin{aligned} f(\varvec\uppi)= & {} \sum \limits _{n=1}^\infty \zeta _n \phi ({\varvec\upxi }_n^{\varvec\uppi}), \end{aligned}$$

where

$$\begin{aligned} \phi \left({ \varvec\upxi }_n^{\varvec\uppi}\right)= & {} E_{\varvec\upxi_n^{\varvec\uppi}}\left( \text{TM}(\bf{Y}) \right) \end{aligned}$$

is the profit of a population with parameter ${\varvec\upxi }_n^{\varvec\uppi}$, and the random vector $\bf{Y}$ is the vector of the phenotype of an animal that is randomly chosen from that population. The breeding policy $\varvec\uppi$ affects the breed’s profit in two ways. It affects the genetic contributions of the selection candidates from the current generation, but also the temporal change of the population’s state from one generation to the next. This dependency, which is illustrated in Fig. 1, is detailed below.

The state ${\varvec\uptheta}_1$ of the population in generation 1 is determined by the vector $\bf{c}_{\varvec\uppi}$ with genetic contributions of the selection candidates, so we can write ${\varvec\uptheta}_1={\varvec\uptheta}_1(\bf{c}_{\varvec\uppi})$. Of course, ${\varvec\uptheta}_1$ depends also on the unknown vectors ${\bf{u}}_i$ with true genetic values of the selection candidates, and on the current state ${\varvec\uptheta}_0$ of the population, but these dependencies are not shown in the formulas.

Since the state of the population changes deterministically, its change from one generation to the next is given by a mapping ${\varvec{\Gamma }}_{\pi }:\Theta \rightarrow \Theta$. That is, the state of the population in generation 2 equals ${\varvec\uptheta}_2^{\varvec\uppi}={\varvec\Gamma }_{\varvec\uppi}({\varvec\uptheta}_1)$, the state in generation 3 is ${\varvec\uptheta}_3^{\varvec\uppi}={\varvec\Gamma }_{\varvec\uppi}(\varvec\uptheta_2)={\varvec\Gamma }_{\varvec\uppi}^2({\varvec\uptheta}_1)$, and so on. In general, ${\varvec\uptheta}_n^{\varvec\uppi}={\varvec\Gamma }_{\varvec\uppi}^{n-1}({\varvec\uptheta}_1)$.

The parameter $\varvec\upxi_n^{\varvec\uppi}$ of the phenotypic distribution in generation *n* can be extracted from the population’s state ${\varvec\uptheta}_n^{\varvec\uppi}$ by a function $\varvec\upxi:\Theta \rightarrow \Xi$, so we can write $\varvec\upxi _n^{\varvec\uppi}=\varvec\upxi ({\varvec\uptheta}_n^{\varvec\uppi})$. Consequently, by writing $\varvec\upxi _{n}^{\varvec\uppi}$ as a function of ${\varvec\uptheta}_1(\bf{c}_{\varvec\uppi})$, the objective function equals $f(\varvec\uppi)=\tilde{f}_{\varvec\uppi}(\bf{c}_{\varvec\uppi})$ with

$$\begin{aligned} \tilde{f}_{\varvec\uppi}({\bf{c}}) = \sum \limits _{n=1}^\infty \zeta _n\> \phi ({\varvec\upxi }_{n}^{\varvec\uppi}({\varvec\uptheta}_1(\bf{c}))). \end{aligned}$$

### The selection index

The purpose of a selection index is that its use to select animals for breeding maximizes genetic gain in the breed’s profit. A selection index should, therefore, predict, how the priorization of a specific animal for breeding increases the value of the objective function, which was defined as the profit over multiple generations. In this section, we first define the aggregate genotype $T_i$ of an animal *i* as a quantity that measures the suitability of that animal for breeding, and we then define the selection index $I_i$ of the animal as the index that has the highest correlation with the unknown aggregate genotype $T_i$.

Prioritizing an animal for breeding causes its genetic contribution $c_i$ to increase beyond some default contribution $\tilde{c}_i$, and decreases the genetic contributions of all other animals from the same sex. The resulting genetic contribution vector is ${\bf{c}}=\tilde{\bf{c}} + {\lambda } \tilde{\bf{e}}_i$, where $\lambda >0$ and $\tilde{\bf{e}}_i={\bf{e}}_i-2\tilde{\bf{c}}_ {{\text{s}}_i}$. The vector ${\bf{e}}_i$ denotes the standard unit vector. Substraction of $2\tilde{\bf{c}}_{\text{s}_i}$ decreases the contributions of the other animals, which ensures that $\bf{c}$ is an admissible contribution vector for $\lambda <\frac{1}{2}$.

As any population should retain a large effective population size, the contributions of single animals are usually low, and the permissible number of offspring of a breeding animal might be restricted. Thus, only small deviations from the default contributions are considered, i.e., the limit $\lambda \rightarrow 0$. The aggregate genotype $T_i$ of an animal *i* is, therefore, defined as the directional derivative of the objective function in the direction of $\tilde{\bf{e}}_i$. That is,

1 $$\begin{aligned} T_i= & {} \lim _{{\lambda }\rightarrow 0}\> \frac{\tilde{f}_{\varvec\uppi}\left( \tilde{\bf{c}} + {\lambda } \tilde{\bf{e}}_i\right) -\tilde{f}_{\varvec\uppi}(\tilde{\bf{c}})}{{\lambda }}. \end{aligned}$$

The vector $\tilde{\bf{c}}$ with default contributions is chosen such that $\tilde{{\varvec\uptheta}}_1=\varvec\uptheta_1(\tilde{\bf{c}})$ is approximately the expected state of the population in the next generation. The value of increasing the genetic contribution of a selection candidate depends on the contributions to be made by other selection candidates, i.e., on the vector $\tilde{\bf{c}}$ with default contributions. However, as pointed out in the background section, it often suffices to define $\tilde{\bf{c}}$ as the vector that assumes equal contributions for all animals of the same sex, in which case $\tilde{{\varvec\uptheta}}_1\approx {\varvec\uptheta}_0$. Although this definition extends the conventional selection index theory, Eq. (1) is certainly not the optimum definition for an aggregate genotype as the choices for $\lambda$ and $\tilde{\bf{c}}$ are somewhat arbitrary. The aggregate genotype has the alternative representation:

$$\begin{aligned} T_i= & {} \frac{\partial \tilde{f}_{\varvec\uppi}}{\partial c_i} (\tilde{\bf{c}})- \nu _{\text{s}_i}, \end{aligned}$$

where the constant $\nu _{\text{s}_i}$ causes $T_i$ to have a mean of zero within each sex. Furthermore, it can be shown that:

$$\begin{aligned} T_{ i}= & {} T_{\tilde{{\varvec\uptheta}}_1}({\bf{u}}_i)\>\>=\>\>{\bf{v}}(\tilde{{\varvec\uptheta}}_1)^{\top } {\bf{u}}_i - \nu _{\text{s}_i}, \end{aligned}$$

where the *L*-vector ${\bf{u}}_i$ with the unknown true genetic values of selection candidate *i* satisfies

$$\begin{aligned} {\bf{u}}_i= & {} \frac{\partial {{\varvec{{\uptheta}}}_{1}}}{\partial c_i}(\tilde{\bf{c}}). \end{aligned}$$

That is, vector ${\bf{u}}_i$ contains the information on animal *i* that would cause the state ${\varvec\uptheta}_1$ of the population in the next generation to change, when the genetic contribution $c_i$ of the animal would deviate from the default contribution $\tilde{c}_i$. The *L*-vector ${\bf{v}}(\tilde{{\varvec\uptheta}}_1)$ is called the vector of the economic weights of the information sources included in ${\bf{u}}_i$. Note that the technical term ‘economic weight’ can be misleading because selection index theory is also applicable to situations that are not economic. Vector ${\bf{v}}(\tilde{{\varvec\uptheta}}_1)$ depends on the actual state of the population, and is the same for all animals. The aggregate genotype is thus a linear function of ${\bf{u}}_i$.

Recall that $\tilde{{\varvec\uptheta}}_1={\varvec\uptheta}_1(\tilde{\bf{c}})$ denotes the population’s state in generation 1 when all the animals from the current generation have their default contributions. Let $\tilde{\varvec\upxi }_n=\varvec\upxi({\varvec\Gamma }_{\varvec\uppi}^{n-1}(\tilde{{\varvec\uptheta}}_1))$ denote the resulting parameter of the phenotypic distribution in generation *n*. The vector with economic weights satisfies:

2 $$\begin{aligned} {\bf{v}}(\tilde{\varvec\uptheta}_1)^\top= & {} \sum \limits _{n=1}^\infty \zeta _n \> \frac{\partial \phi }{\partial {\varvec\upxi }_n^{\varvec\uppi}} (\tilde{\varvec\upxi }_n) \cdot \frac{\partial \varvec\upxi_n^{\varvec\uppi}}{\partial {\varvec\uptheta}_1} (\tilde{\varvec\uptheta}_1), \end{aligned}$$

where we used the naming convention that the derivative of a vector by a vector is the matrix with partial derivatives, and “$\cdot$” denotes the matrix product. The *n*^th^ summand quantifies how a change of the population’s state in generation 1 would cause a change of the breed’s profit in generation *n*.

The selection index $I_i$ of animal *i* is defined by a linear function that has the highest correlation with the unknown aggregate genotype $T_i$. That is,

$$\begin{aligned} I_i={\bf{b}}_i^\top \hat{\bf{u}}_i \>\> \>\>\text{with}\>\> \>\>{\bf{b}}_i={\bf{P}}_{i}^{-1}{\bf{G}}_i \>{\bf{v}}(\tilde{\varvec\uptheta}_1), \end{aligned}$$

where ${\bf{G}}_i=\text{Cov}(\hat{\bf{u}}_i,{\bf{u}}_i)$ is the matrix with covariances between estimated and true genetic values of animal *i*, and ${\bf{P}_{i}}=\text{Cov}(\hat{\bf{u}}_i)$ is the covariance matrix of the estimated genetic values. The covariance matrices depend on the class to which animal *i* belongs. In practice, this means that animals with genomic estimated breeding values and animals with conventional estimated breeding values have different index weights. However, in the special case that model-based covariance matrices are used and the information sources are all best linear unbiased predictions (BLUP) of breeding values obtained from a single multiple-trait model, then ${\bf{G}}_i={\bf{P}}_{i}$, and ${\bf{b}}_i={\bf{v}}(\tilde{\varvec\uptheta}_1)$. In general, the information sources in the selection index and the aggregate genotype do not need to be identical.

### The combining ability

Although the selection index is defined under the assumption of random mating, in practice, index selection is applied to non-random mating populations. A common method of mate selection relies on the expected combining ability of a female with a potential mating partner [17]. The true combining ability $T_{ij}=T_{{\varvec\uptheta}_0}({\bf{u}}_i, {\bf{u}}_j)$ between two potential mating partners is the expected total merit of their offspring. As the true combining ability is unknown, the combining ability could, for example, be estimated by the function $C_{{\varvec\uptheta}_0}(\hat{\bf{u}}_i,\hat{\bf{u}}_j)$ that has the highest correlation with the true combining ability. However, as $T_{ij}$ is not a linear function of ${\bf{u}}_i$ and ${\bf{u}}_j$, it is not obvious what this function is. For convenience, one could choose $C_{{\varvec\uptheta}_0}(\hat{\bf{u}}_i,\hat{\bf{u}}_j)=T_{{\varvec\uptheta}_0}(\hat{\bf{u}}_i, \hat{\bf{u}}_j)$ as an approximation.

### Application to specific merit functions

#### Multiple breeding objectives

It is common for a breed to have multiple breeding objectives. In livestock farming, these objectives include increasing the economic profit of the farmers [18], reducing the ecological footprint of the animals per unit of food produced [19], improving animal welfare [20], matching farmer preferences [21], and accounting for the consumer’s willingness to pay for specific desires [22]. Breeding programs for companion animals have the main objectives to improve the attractiveness of the breeds for their owners, and to improve health and longevity. It is often feasible to calculate an animal’s merit for each breeding objective separately by using appropriate methodology. This provides a partial merit function $\text{PM}_o:\mathbb {R}^K\rightarrow \mathbb {R}$ for each breeding objective *o*, where *K* is the number of traits. The different partial merits can then be combined into the animal’s total merit as:

$$\begin{aligned} \text{TM}({\bf{y}}_{i})= & {} \sum \limits _{o=1}^O w_o \text{PM}_{o}({\bf{y}}_{i}), \end{aligned}$$

where *O* is the number of different breeding objectives, $w_o\ge 0$ is the importance placed on the breeding objective *o*, and $\sum _o w_o=1$. Note that it is permissible, but not required, that the different partial merit functions depend on different sets of traits. Relevant for the design of breeding programs is the profit function of the breed:

3 $$\begin{aligned} \phi (\varvec\upxi )= & {} \sum \limits _{o=1}^O w_o \phi _o(\varvec\upxi ), \end{aligned}$$

which depends on the breed’s profit with respect to a given breeding objective *o*:

$$\begin{aligned} \phi _o(\varvec\upxi )= & {} E_{\varvec\upxi } \left( \text{PM}_o(\bf{Y})\right) . \end{aligned}$$

Thereby, $\bf{Y}$ is the random *K*-vector with trait measurements of an animal that is chosen from a population with parameter $\varvec\upxi$. The vector with economic weights satisfies:

$$\begin{aligned} {\bf{v}}(\tilde{\varvec\uptheta}_1)= & {} \sum \limits _{o=1}^O w_o {\bf{v}}_o(\tilde{\varvec\uptheta}_1), \end{aligned}$$

where ${\bf{v}}_o(\tilde{\varvec\uptheta}_1)$ can be called the vector with economic weights for breeding objective *o*. The equation for computation can be obtained from Eq. (2) by substituting ${\bf{v}}(\tilde{\varvec\uptheta}_1)$ with ${\bf{v}}_o(\tilde{\varvec\uptheta}_1)$ and $\phi$ with $\phi _o$. The partial aggregate genotype $\text{PT}_{oi}={\bf{v}}_o(\tilde{\varvec\uptheta}_1)^{\top } {\bf{u}}_i$ for breeding objective *o* quantifies how the use of animal *i* for breeding improves the match of the breed to the breeding objective *o* in future generations.

#### Distance-based merit functions

This section also considers multiple breeding objectives and assumes a certain functional form for the partial merit functions. Animal breeders often have strong beliefs about the optimum phenotype, and consumers often have strong beliefs about the optimum taste of the animal’s products. Moreover, profit calculations and the law of diminishing marginal returns often dictate a specific nonlinear form of a partial merit function. Selection for increased performance could lead to unfavorable genetic correlations and thus to an erosion of the genetic variance in the index. This would cause the trait mean to converge towards some optimum even if the functional form of the true profit function does not have a maximum. In all these cases, the true profit function could be replaced for simplicity by an approximate profit function that depends only on the weights $\omega _{ok}$ of the traits *k* and their presumed optimum values $\text{Opt}_{ok}$. This section applies the general theory to this important special case by assuming normally distributed phenotypes.

Suppose that a distance is defined on the space $\mathbb {R}^K$ of phenotypes such that the distance $d_o({\bf{y}}_{i}, {\bf{y}}_j)$ between two animals *i* and *j* is small, if they are similar with respect to all traits that are relevant to breeding objective *o*. The partial merit for breeding objective *o* is then defined as:

$$\begin{aligned} \text{PM}_{o}({\bf{y}}_{i})= & {} \tau _\text{max}-d_o({\bf{y}}_{i}, {\bf{Opt}}_o), \end{aligned}$$

with an arbitrary constant $\tau _\text{max}$. An animal with partial merit $\text{PM}_{o}({\bf{y}}_{i})=\tau _\text{max}$ fits the breeding objective *o* perfectly, while ordinary specimens of the breed have a partial merit smaller than $\tau _\text{max}$. The distance could be defined by the so-called weighted Manhattan norm [23]:

$$\begin{aligned} d_o({\bf{y}}_{i}, {\bf{y}}_j)= & {} \sum _{k=1}^K \omega _{ok} \left| y_{ik}-y_{jk} \right| , \end{aligned}$$

where $\omega _{ok}\ge 0$ is the weight that the breeding objective *o* places on trait *k*. Thus, the partial merit function equals:

4 $$\begin{aligned} \text{PM}_o({\bf{y}}_{i})= & {} \tau _\text{max}- \sum _{k=1}^K \omega _{ok} \left| y_{ik}-\text{Opt}_{ok} \right| . \end{aligned}$$

The Manhattan norm has the advantage that the associated partial merit is a piecewise linear function. In particular, if all trait optima are outside the phenotypic range of the breed, then the partial merit function is linear on the relevant domain. Hence, classical selection index theory can be applied for maximizing short-term genetic gain.

The profit of the breed is obtained by integrating the partial merit functions over the distribution of phenotypes. Most traits are additively affected by many QTL with small effects. Thus, the central limit theorem states that they are approximately normally distributed. That is, ${\bf{Y}} \sim \mathcal {N}_{K}( {\varvec\upmu },\varvec{\Sigma }_\text{P})$, where ${\varvec\upmu }$ is the *K*-vector of the trait means, and $\varvec{\Sigma }_\text{P}$ is the $K \times K$-phenotypic covariance matrix. We have:

$$\begin{aligned} Y_{k}-\text{Opt}_{ok}\sim & {} \mathcal {N}( \mu _k-\text{Opt}_{ok},\sigma _{\text{P}k}^2), \end{aligned}$$

where $\sigma _{\text{P}k}^2=\Sigma _{\text{P}kk}$ is the phenotypic variance of trait *k*. In general, for a normally distributed random variable $X\sim N(\mu ,\sigma ^2)$, the random variable |*X*| has a folded normal distribution with mean:

$$\begin{aligned} \psi (\mu ,\sigma )= & {} \sigma \sqrt{\frac{2}{\pi }} \>\text{exp}\left( \frac{-\mu ^2}{2\sigma ^2 }\right) +\mu \left( 1-2\Phi \left( \frac{-\mu }{\sigma }\right) \right) , \end{aligned}$$

where $\Phi$ is the cumulative distribution function of the standard normal distribution. Consequently, the profit of the breed with respect to breeding objective *o* equals:

5 $$\begin{aligned} \phi _o(\xi )= & {} \tau _\text{max} - \sum _{k=1}^K \omega _{ok} \>\psi (\mu _{k}-\text{Opt}_{ok},\sigma _{\text{P}k}). \end{aligned}$$

Equations (3) and (5) show that the profit of a breed depends on the *K*-vector ${\varvec\upmu }$ with trait means, and on the *K*-vector $\varvec{\upsigma }_\text{P}$ with phenotypic standard deviations, but not on the genetic correlations. Therefore, we can define the parameter space $\Xi$ as consisting of all tuples $\varvec\upxi =({\varvec\upmu }, \varvec\upsigma _{\text{P}})$. The assumptions about the partial merit functions impose a specific structure on the economic weights. The vector with economic weights for breeding objective *o* has the following representation:

$$\begin{aligned} {\bf{v}}_o(\tilde{\varvec\uptheta}_1)= & {} \sum _{k=1}^K \omega _{ok} \> {\bf{v}}_k(\tilde{\varvec\uptheta}_1), \end{aligned}$$

where vector ${\bf{v}}_k(\tilde{\varvec\uptheta}_1)$ is the same for all breeding objectives. In classical selection index theory, $\omega _{ok}$ would be the economic weight of trait *k* for breeding objective *o*, and vector ${\bf{v}}_k(\tilde{\varvec\uptheta}_1)$ would be identical with the unit vector ${\bf{e}}_k\in \mathbb {R}^K$. In the general case considered here, the *L*-vector ${\bf{v}}_k(\tilde{\varvec\uptheta}_1)$ contains the contributions that the different information sources in vector ${\bf{u}}_i$ make to the improvement of trait *k*. The information sources could improve the trait by shifting the trait mean towards the optimum, or by reducing the phenotypic variance. The vector ${\bf{v}}_k(\tilde{\varvec\uptheta}_1)$ can indeed be decomposed as:

$$\begin{aligned} {\bf{v}}_k(\tilde{\varvec\uptheta}_1)= & {} {\bf{v}}_k^{\text{m}}(\tilde{\varvec\uptheta}_1) + {\bf{v}}_k^{\text{v}}(\tilde{\varvec\uptheta}_1), \end{aligned}$$

where ${\bf{v}}_k^{\text{m}}(\tilde{\varvec{{\uptheta}}}_1)$ contains the contributions that the information sources have to the improvement of the trait by adjusting the mean, and vector ${\bf{v}}_k^{\text{v}}(\tilde{\varvec{{\uptheta}}}_1)$ contains the contributions that the information sources have to the improvement of the trait by adjusting the phenotypic variance of trait *k*. The contribution vectors have the following representations:

6 $$\begin{aligned} {\bf{v}}_k^{\text{m}}(\tilde{\varvec\uptheta}_1)^\top= & {} \sum \limits _{n=1}^\infty \zeta _n \> \chi _{\text{m}}^{k}(\tilde{\varvec\upxi }_n)\> \frac{\partial {\mu }_{nk}}{\partial {\varvec\uptheta}_1} (\tilde{\varvec\uptheta}_1),\\ {\bf{v}}_k^{\text{v}}(\tilde{\varvec\uptheta}_1)^\top= & {} \sum \limits _{n=1}^\infty \zeta _n \>\chi _{\text{v}}^k(\tilde{\varvec\upxi }_n) \frac{\partial \sigma _{\text{P}nk}}{\partial {\varvec\uptheta}_1} (\tilde{\varvec\uptheta}_1),\nonumber \end{aligned}$$

where the row-vectors $\frac{\partial {\mu }_{nk}}{\partial {\varvec\uptheta}_1} (\tilde{\varvec\uptheta}_1)$ and $\frac{\partial \sigma _{\text{P}nk}}{\partial {\varvec\uptheta}_1} (\tilde{\varvec\uptheta}_1)$ quantify how a change of the population’s state in generation 1 would cause the mean and standard deviation of trait *k* to change in generation *n*. These vectors depend on the underlying genetic model and on the selection index that will be used in future generations. The weight

7 $$\begin{aligned} \chi _{\text{m}}^{k}(\tilde{\varvec\upxi }_n)= & {} 2\Phi \left( \frac{\text{Opt}_{ok}-\tilde{\mu }_{nk}}{\tilde{\sigma }_{\text{P}nk}}\right) -1 \>\>\approx \>\> \text{sign}(\text{Opt}_{ok}-\tilde{\mu }_{nk}) \end{aligned}$$

quantifies the extent to which increasing the mean of trait *k* in generation *n* would be desirable. The weight approaches 0 when the trait mean in generation *n* is close to the optimum, and $\pm 1$ when the trait mean is several phenotypic standard deviations away. A further shift in the trait mean in the direction of the optimum is therefore particularly advantageous if the trait mean deviates considerably from the optimum. In particular, the approximation holds only if the trait mean differs by at least two phenotypic standard deviations from the optimum. The weight

8 $$\begin{aligned} \chi _{\text{v}}^k(\tilde{\varvec\upxi }_n)= & {} - \sqrt{\frac{2}{\pi }} \>\text{exp}\left( -\frac{1}{2} \frac{(\text{Opt}_{ok}-\tilde{\mu }_{nk})^2}{\tilde{\sigma }_{\text{P}nk}^2}\right) \end{aligned}$$

is always negative, which implies that only a reduction of the phenotypic variance in generation *n* could improve the profit in that generation. Of course, increasing the variance could improve the profit in later generations because it could lead to an increased selection response in later generations. The factor on the right-hand side approaches 1 when the trait mean in generation *n* is close to the optimum, but 0 when the trait mean is several phenotypic standard deviations away. Hence, decreasing the phenotypic variance is only advantageous if the trait mean is already close to the optimum.

The presumed functional form of the partial merit functions also enables exact formulas for the combining ability. The true combining ability $T_{ij}$ of a male *i* with a female *j*, which was defined as the expected total merit of the offspring from the mating, can be calculated as:

$$\begin{aligned} T_{ij}= & {} \sum \limits _{o=1}^O w_o \text{PT}_{ijo}, \end{aligned}$$

where the partial combining ability of male *i* with female *j* with respect to breeding objective *o* equals:

$$\begin{aligned} \text{PT}_{ijo}= & {} \tau _\text{max} - \sum \limits _{k=1}^K \omega _{ok}\> \psi (\mu _{ijk}-\text{Opt}_{ok},\sigma _{ijk}). \end{aligned}$$

For the additive genetic model, the mean of trait *k* in the offspring equals:

$$\begin{aligned} \mu _{ijk}= & {} \mu _{k} + \frac{\text{TBV}_{ik}+\text{TBV}_{jk}}{2}, \end{aligned}$$

and the variance of trait *k* in the offspring is:

$$\begin{aligned} \sigma _{ijk}^2=\text{MV}_{ik}+\text{MV}_{jk}+\sigma _{Ek}^2, \end{aligned}$$

where $\sigma _{Ek}^2$ is the environmental variance, $\mu _k$ is the mean of trait *k* in the base population, and $\text{MV}_{ik}$ is the Mendelian sampling variance of animal *i* for trait *k*. Thereby, the Mendelian sampling variance of an animal is defined as the variance of the true breeding values of the haplotypes that are transmitted by the animal to its offspring. Note that for the additive genetic model, two animals have a good combining ability if the breeding value of their offspring is close to the optimum, while for a model with additive and dominance effects, the presence of a heterosis effect in the offspring can also contribute to a good combining ability.

### Application to specific genetic models

This section provides insight into the genetic information on the selection candidates that might influence their optimum selection indices. First, a simple genetic model is considered that provides the commonly used definition of an aggregate genotype, and second, the potentially relevant information on the genetic value of a selection candidate is identified based on more realistic genetic models.

#### Simple genetic model

Let us assume that the total merit function is distance-based and piecewise linear, the phenotypes have a multivariate normal distribution, phenotypic and genetic variances and covariances are constants, the trait optima are several phenotypic standard deviations away from the trait means, and the same selection index will be used in all future generations until $\zeta _n$ approaches 0. It can be shown that under these simplifying assumptions, the aggregate genotype $\text{PT}_{oi} = {\bf{v}}_o(\tilde{\varvec\uptheta}_1)^\top {\bf{u}}_i$ for breeding objective *o* has the well-known representation:

$$\begin{aligned} \text{PT}_{oi}= & {} \sum _{k=1}^K v_{ok} \text{TBV}_{ik}, \end{aligned}$$

where $v_{ok}=\pm \omega _{ok}$. A full proof is given in Additional file 1, but an outline of the proof is provided here. As the genetic variances are constants, $\frac{\partial \sigma _{\text{P}nk}}{\partial {\varvec\uptheta}_1} (\tilde{\varvec\uptheta}_1)=0$ in Eq. (6). The state of a population is described only by the vector with trait means, so ${\varvec\uptheta}_n={{\varvec\upmu }}_n$. As the trait optima are several phenotypic standard deviations away from the trait means, we have approximately $\chi _{\text{m}}^{k}(\tilde{\varvec\upxi }_n)=\pm 1$ and $\chi _{\text{v}}^{k}(\tilde{\varvec\upxi }_n)=0$. If the same selection index is used in each generation, then ${{\varvec\upmu }}_n={{\varvec\upmu }}_1+(n-1) \varvec{\Delta \upmu }$, where the change $\varvec{\Delta \upmu }$ of the population mean from one generation to the next is a constant. Consequently, $\frac{\partial {\mu }_{nk}}{\partial {\varvec\uptheta}_1} (\tilde{\varvec\uptheta}_1)=\frac{\partial {\mu }_{1k}}{\partial {{\varvec\upmu }}_1} (\tilde{{\varvec\upmu }}_1)={\bf{e}}_{k}$ is the unit vector in Eq. (6), so ${\bf{v}}_k^{\text{m}}(\tilde{\varvec\uptheta}_1)=\pm {\bf{e}}_{k}$. On the other hand, ${\bf{v}}_k^{\text{v}}(\tilde{\varvec\uptheta}_1)=\varvec{0}$. The vector containing the contributions of the different information sources to improve trait *k* thus equals ${\bf{v}}_k(\tilde{\varvec\uptheta}_1)= \pm {\bf{e}}_{k}$. That is, only the TBV of trait *k* contributes to the improvement of trait *k*. Consequently, the $k$^th^ component of vector ${\bf{v}}_o(\tilde{\varvec\uptheta}_1)$ is $v_{ok}=\pm \omega _{ok}$. The claim follows by noting that ${\bf{u}}_i=\frac{\partial {{\varvec\upmu }_{1}}}{\partial c_i}(\tilde{\bf{c}}) = {\bf{TBV}}_{i}$ is the *K*-vector with the true breeding values of animal *i*.

#### General additive model

Prioritizing an animal for breeding can affect the profit of the population in a future generation *n* by changing the parameter $\varvec\upxi _{n}=\varvec\upxi _{n}^{\varvec\uppi}$ of the phenotypic distribution in that generation. The expected change of vector $\varvec\upxi _{n}$ that results from a deviation of the animal’s genetic contribution from its default contribution $\tilde{c}_i$ equals the directional derivative of $\varvec\upxi _{n}$ at $\tilde{\bf{c}}$ in the direction of $\tilde{\bf{e}}_i$, which is:

$$\begin{aligned} \lim _{{\lambda }\rightarrow 0}\> \frac{\varvec\upxi _{n}\left( \tilde{\bf{c}} + {\lambda } \tilde{\bf{e}}_i\right) -\varvec\upxi _{n}(\tilde{\bf{c}})}{{\lambda }}= & {} \frac{\partial \varvec\upxi _{n}}{\partial c_i}(\tilde{\bf{c}}) - 2 \tilde{\bf{c}}_{s_i}^\top \frac{\partial \varvec\upxi _{n}}{\partial \bf{c}}(\tilde{\bf{c}}). \end{aligned}$$

This equation depends on animal *i* only via vector $\frac{\partial \varvec\upxi _{n}}{\partial c_i}(\tilde{\bf{c}})$. Consequently, the information on a selection candidate that determines its value for breeding is contained in that vector. The vector $\varvec\upxi _{n}$ provides the trait means and the phenotypic variances in generation *n*, so the components of the vector are given by $\frac{\partial \mu _{nk}}{\partial c_i}(\tilde{\bf{c}})$ and $\frac{\partial {\sigma _{\text{P}nk}^2}}{\partial c_i}(\tilde{\bf{c}})$. The relevant information on the animal that is identified by calculating these derivatives for generations $n=1,2$ is then put into vector ${\bf{u}}_i$. In a second step, the population parameters in vector ${\varvec\uptheta}_{n}$ are identified that satisfy the equation ${\bf{u}}_i=\frac{\partial {\varvec\uptheta}_{1}}{\partial c_i}(\tilde{\bf{c}})$ for all animals *i*. Deriving recursion equations for computing ${\varvec\uptheta}_{n+1}$ from ${\varvec\uptheta}_{n}$ is beyond the scope of this paper. In summary, the application of selection index theory to traits with a specific genetic architecture requires the identification of the information ${\bf{u}}_i$ on the selection candidate that can affect the animal’s aggregate genotype, and the identification of the corresponding population parameter ${\varvec\uptheta}_1(\tilde{\bf{c}})$ for which the vector with partial derivatives equals ${\bf{u}}_i$ for all animals *i*. In this section, the general theory is applied to a general additive model to determine what this information is. For simplicity, random mating is assumed in the population during the next generations. We have:

$$\begin{aligned} \frac{\partial {\mu _{1k}}}{\partial c_i}(\tilde{c})= & {} \text{TBV}_{ik}. \end{aligned}$$

Hence, the information on animal *i* that causes the mean of trait *k* in the next generation to change is its breeding value. We now identify the information on the animal that could lead to a change of the phenotypic variance. It is assumed that the genetic value of an animal is only affected by additive genetic effects, so $\varvec\upsigma _{\text{G}}^2=\varvec\upsigma _{\text{A}}^2$. Furthermore, the environmental variances included in vector $\varvec\upsigma _\text{E}^2$ are assumed to be constant over time. Consequently, the derivative of the phenotypic variance with respect to $c_i$ is equal to the derivative of the additive variance, which is:

$$\begin{aligned} \frac{\partial {\sigma _{\text{A}nk}^2}}{\partial c_i}(\tilde{\bf{c}})= & {} \frac{\partial {\sigma _{\text{PA}nk}^2}}{\partial c_i}(\tilde{\bf{c}}) + \frac{\partial {\sigma _{\text{MT}nk}^2}}{\partial c_i}(\tilde{\bf{c}}) \end{aligned}$$

in generation *n* for trait *k*. Thereby, $\sigma _{\text{PA}nk}^2$ is the variance of the parent average of an animal that is randomly chosen from generation *n* and the parent average of an animal is defined as the mean of the true breeding values of its sire and dam. The value $\sigma _{\text{MT}nk}^2$ is the variance of the Mendelian sampling terms that were received by an animal that is randomly chosen from generation *n*. The information on animal *i* that causes the variance of the parent average of trait *k* in the next generation to change is given by:

$$\begin{aligned} \frac{\partial \sigma _{\text{PA}1k}^2}{\partial c_i}(\tilde{\bf{c}})= & {} \frac{1}{2} \left( \text{TBV}_{ik} - m_{s_ik}\right) ^2 - \frac{m_{s_ik}^2}{2}, \end{aligned}$$

where $m_{s_ik}=2\tilde{\bf{c}}_{\text{s}_i}^\top {\bf{TBV}}_{k}$ is the mean breeding value of breeding animals of the same sex. The second term is a constant and can be neglected, while the first term is inversely related to the animal’s contribution to the Bulmer effect [24]. That is, if the animal’s breeding value deviates little from the average breeding value of the other selected parents, then prioritizing the animal for breeding would decrease the variance of the parent average in the next generation. Expanding the equation shows that the relevant information on the animal is its true breeding value $\text{TBV}_{ik}$ and its squared true breeding value $\text{TBV}_{ik}^2$. We have:

$$\begin{aligned} \frac{\partial {\sigma _{\text{MT}1k}^2}}{\partial c_i}(\tilde{c})= & {} 2\text{MV}_{ik}. \end{aligned}$$

Thus, the information on the animal that could cause the variance $\sigma _{\text{MT}nk}^2$ of the Mendelian sampling terms for trait *k* in the next generation $n=1$ to change is the animal’s true Mendelian sampling variance $\text{MV}_{ik}$. The use of an animal for breeding affects not only the genetic variance in the next generation, but also the genetic variance in the upper-next generation $n=2$. By assuming that the contribution of the animal to the next generation is equal to its contribution to the upper-next generation, we have:

$$\begin{aligned} \frac{\partial {\sigma _{\text{MT}2k}^2}}{\partial c_i}(\tilde{\bf{c}})= & {} 2E(\text{MV}_{o_ik}),\nonumber \end{aligned}$$

where $o_i$ is a randomly chosen offspring of animal *i*. Thus, the optimum selection index could depend not only on the breeding values of the selection candidates, but also on the squared breeding values, and on the expected Mendelian sampling variances of the animal and its offspring. However, the most suitable parameters for inclusion in the model might not be the Mendelian sampling variances themselves, but parameters that are suitable to calculate these variances. These parameters depend on the presumed genetic architecture of the trait. In the following, a polygenic additive model, and a QTL-based additive model with a polygenic term are considered.

#### Polygenic additive model

The polygenic additive model assumes that the breeding values are affected by a large number of QTL and that the effects of single QTL are so small that they can be neglected. In this case, the expected Mendelian sampling variances of the haplotypes that are transmitted by animal *i* and its offspring $o_i$ equal:

$$\begin{aligned} \text{MV}_{ik}= & {} \frac{1-F_i}{4} \tilde{\sigma }_{Ak}^2, \>\>\text{and}\\ E(\text{MV}_{o_ik})= & {} \frac{1-\overline{f}_i}{4} \tilde{\sigma }_{Ak}^2, \end{aligned}$$

respectively, where $F_i$ is the inbreeding coefficient of animal *i*, and $\overline{f}_i=\overline{f}_i(\bf{c})$ is the average kinship of animal *i* with breeding animals of the opposite sex. The corresponding population parameters with partial derivatives at $\tilde{\bf{c}}$ being $2\overline{f}_i(\tilde{\bf{c}})$ and $F_i$, respectively, are the average inbreeding coefficient in generation 1:

$$\begin{aligned} \overline{F}_1(\bf{c})= & {} \sum _i {c}_i \overline{f}_i(\bf{c}), \end{aligned}$$

and the average inbreeding coefficient in the parents of generation 1:

$$\begin{aligned} \overline{F}_1^P(c)= & {} \sum _i c_i F_i. \end{aligned}$$

Hence, the number of model parameters can be greatly reduced by including $2\overline{f}_i$ and $F_i$ in vector ${\bf{u}}_i$, rather than $\text{MV}_{i1},...,\text{MV}_{iK}$ and $E(\text{MV}_{o_i1}),...,E(\text{MV}_{o_iK})$. This leads to the following model parameterization:

9 $$\begin{aligned} {\varvec\uptheta}_n\>=\> \left( \begin{array}{c} {{\upmu }}_{n}\\ \varvec\upsigma _{\text{PA}n}^2 \\ \overline{F}_n\\ \overline{F}_n^P\\ \end{array}\right) ,{} & {} {\bf{u}}_i\>=\>\left( \begin{array}{c} \bf{TBV}_{i}\\ \frac{1}{2}({\bf{TBV}}_{i}-\varvec{m}_{s_i})^2\\ 2\overline{f}_i\\ F_i \end{array}\right) , \end{aligned}$$

where the square is taken component-wise and the constant terms were omitted in vector ${\bf{u}}_i$. Most model parameters satisfy recursion equations that depend on the selection index that will be used in future generations. The computation of the recursion equations, however, is not part of the present paper. Instead the theory is applied in a simulation study that estimates the index weights directly. This model could for example be used to determine the appropriate weight of the kinship coefficient $\overline{f}_i$ in the selection index when a population is under directional selection. A negative weight would favor outcross animals for breeding and could thus increase the genetic variance and genetic gain in future generations. Directional selection is obtained as the limiting case in which $\chi _{\text{m}}^k(\tilde{\varvec\upxi }_n)= \pm 1$ and $\chi _{\text{v}}^k(\tilde{\varvec\upxi }_n)= 0$ in Eq. (6). This model also arises as a special case of the model below in which QTL with non-negligible effects can be present.

#### QTL-based additive model with polygenic term

In the QTL-based additive model with polygenic term, the true breeding value $\text{TBV}_{ik}$ of animal *i* is affected by a polygenic breeding value $\text{TBV}_{ik}'$ and the effects $a_{qk}$ of large QTL. Thus,

10 $$\begin{aligned} \text{TBV}_{ik}= & {} \text{TBV}_{ik}' + \sum _{q=1}^Q (X_{iq}-2p_{0q})a_{qk}, \end{aligned}$$

where $X_{iq}\in \{0,1,2\}$ is the allele content of animal *i* for QTL *q* and $p_{nq}$ is the frequency of the alternative allele of QTL *q* in generation *n*. The previous model arises as a special case when $Q=0$ and $\text{TBV}_{ik} = \text{TBV}_{ik}'$. The expected Mendelian sampling variances of animal *i* and its offspring $o_i$ are:

$$\begin{aligned} \text{MV}_{ik}=\, & {} \frac{1-F_i}{4} \tilde{\sigma }_{Ak}^2 + \sum _{q=1}^Q \frac{H_{iq}}{4} a_{qk}^2, \>\>\>\>\text{and}\\ E(\text{MV}_{o_ik})= \, & {} \frac{1-\overline{f}_i}{4} \tilde{\sigma }_{Ak}^2 + \sum _{q=1}^Q \frac{E(H_{o_iq})}{4} a_{qk}^2,\nonumber \end{aligned}$$

respectively, where $H_{iq}\in \{0,1\}$ equals one if animal *i* is heterozygous at QTL *q*, and

$$\begin{aligned} E(H_{o_iq})= \,& {} \frac{X_{iq}}{2}\Big (1-p_{1q}^{\overline{\text{s}}_i}({\bf{c}})\Big )+p_{1q}^{\overline{\text{s}}_i}({\bf{c}})\left( 1-\frac{X_{iq}}{2}\right) \end{aligned}$$

is the expected heterozygosity of an offspring of animal *i* at QTL *q*. Thereby, $p_{1q}^{\overline{\text{s}}_i}(\bf{c})$ is the frequency of the alternative allele of QTL *q* in the haplotypes from generation 1 that are received from the parents of the opposite sex. In a random mating population, this value does not depend on animal *i*. Hence, the expected heterozygosity $E(H_{o_iq})$ of the offspring at QTL *q* is affected by animal *i* only via its allele content $X_{iq}$. The corresponding population parameters with partial derivatives at $\tilde{\bf{c}}$ being $\frac{X_{iq}}{2}$ and $H_{iq}$, respectively, are the frequency

$$\begin{aligned} p_{1q}({\bf{c}})= & {} \sum _i c_i \frac{X_{iq}}{2} \end{aligned}$$

of QTL *q* in generation 1 and the average heterozygosity

$$\begin{aligned} \overline{H}_{1q}^P({\bf{c}}) = \sum _i c_i H_{iq} \end{aligned}$$

of QTL *q* in the parents of generation 1. Thus, appropriate model parameters can be obtained by appending the allele frequencies $p_{n1},...,p_{nQ}$ and the allele heterozygosities $\overline{H}_{n1}^P,...,\overline{H}_{nQ}^P$ to vector ${\varvec\uptheta}_n$, and by appending the standardized allele contents $\frac{X_{i1}}{2},..,\frac{X_{iQ}}{2}$ and the indicators for heterozygosity $H_{i1},...,H_{iQ}$ to vector ${\bf{u}}_i$ in Eq. (9).

## Examples

This section applies selection index theory to an illustrative example with a single breeding objective and one trait. The scenario reflects a situation in which conventional selection towards the optimum causes the frequencies of large QTL to move towards intermediate values, which would maintain an undesired large phenotypic variance. The example resembles a situation in dog breeding, where QTL with large effects on the phenotypes can be present.

It is assumed that the trait is affected by two known large QTL with equal effects that both segregate at an allele frequency of $p_{01}\approx p_{02}\approx 0.1$ in generation 0. The trait has a polygenic variance of $\tilde{\sigma }_{\text{A}}^2=0.3$, and an environmental variance of $\sigma _{\text{E}}^2=0.7$. The trait mean for genotype $(X_{i1},\>X_{i2})=( 0, \> 0 )$ is 0 in generation 0 and the trait optimum equals $\text{Opt}=2$. As the additive effects of both QTL equal $a_{1}=a_{2}=1$, at most, one of the QTL would be needed to reach the optimum.

As shown in the previous sections, the aggregate genotype is in general a linear function of the true breeding value $\text{TBV}_{i}$, the squared breeding value $\text{TBV}_{i}^2$, the inbreeding coefficient $F_i$, the animal’s kinship with the population $\overline{f}_i$, the allele contents $X_{i1},X_{i2}$, and the indicators $H_{i1},H_{i2}$ for heterozygosity, which are appropriately standardized and included in vector ${\bf{u}}_i$. The selection index has the representation $I_i={\bf{b}}^\top \hat{\bf{u}}_i$, where the vector ${\bf{b}}={\bf{b}}({\varvec\uptheta}_0)$ with weights given to the different information sources depends on the current state ${\varvec\uptheta}_0$ of the population.

Instead of first computing the economic weights with Eq. (6), and computing the weights of the selection index thereafter, we estimated the weights directly in a simulation study. A random mating population with 1000 animals was simulated, which were selected for nine generations by index selection. In each generation, the 50% animals with the highest selection index were selected for breeding. In the simulation, each breeding animal had four offspring. Hence, a similar amount of information was available on all animals. Vector ${\bf{b}}$, therefore, was the same for all animals.

Breeding values were estimated with the mixed linear model $y_i=\mu +a_{i}+e_i$, where the residuals $e_i$ are independent and identically distributed, and the true breeding values $a_{i}=\text{TBV}_{i}' + \sum _{q=1}^2 (X_{iq}-2p_{0q})a_{q}$ satisfy the assumptions of the QTL-based additive model with polygenic term. The vector with polygenic breeding values has a multivariate normal distribution $\textbf{TBV}' \sim N(\textbf{0}, \sigma _{a'}^2 \textbf{A})$ with covariance matrix proportional to the additive relationship matrix $\textbf{A}$. The breeding values were estimated from two-generation pedigrees, and the selection program was preceded by two generations without selection in order to get pedigree data for all animals.

Some constraints were applied on the coefficients of the selection index. First, the weights for $F_i$ and $\overline{f}_i$ were constrained to be zero because the optimum index would give positive weights to these parameters when the trait has reached the optimum as this would decrease the phenotypic variance. This artefact arises because no trait under directional selection is included in the index, and because the underlying additive model does not account for inbreeding depression. The selection index has thus the following representation:

$$\begin{aligned} I_i= \, & {} b_{1} \text{EBV}_{i} + b_{2} \frac{1}{2} (\text{EBV}_{i}-\hat{m}_{s_i})^2 + b_{3} \frac{X_{i1}}{2} + b_{4} \frac{X_{i2}}{2} + b_{5} H_{i1}+ b_{6} H_{i2}. \end{aligned}$$

For simplicity, we assumed constant weights as long as the trait was under directional selection, and we used another set of weights when the trait was under stabilizing selection. During stabilizing selection, only the weights $b_3$ to $b_6$ were constant, while the weights $b_1$ and $b_2$ depended on the actual state of the population as:

$$\begin{aligned} b_1= \,& {} \frac{(\text{Opt} - \hat{\mu }-\hat{m}_{s_i})}{1+|\text{Opt} - \hat{\mu }-\hat{m}_{s_i}|} \alpha ,\\ b_2= & {} - \frac{1}{1+|\text{Opt} - \hat{\mu }-\hat{m}_{s_i}|} \alpha , \end{aligned}$$

where the estimated paramter $\alpha$ was kept constant. This restriction ensured that the genetic value of animals that had a maximum index was equal to the optimum. Consequently, the trait mean converged towards the optimum once the QTL alleles were fixed. Hence, ${\bf{b}}\in \{{\bf{b}}_{\text{d}}, {\bf{b}}_{\text{s}}\}$, where vector ${\bf{b}}_{\text{d}}$ was used for directional selection and ${\bf{b}}_{\text{s}}={\bf{b}}_{\text{s}}({\boldsymbol{{\uptheta}}})$ was used for stabilizing selection. Directional selection ended when the mean trait value in the next generation was expected to surpass the optimum.

The optimum selection index was searched with a hill climbing algorithm, whereby the objective was to maximize the average profit of the population in generations 1 to 9. The value of the estimated objective function was averaged over 200 replicates.

A period of directional selection was followed by a period of stabilizing selection. Table 2 shows the optimum index weights of the different information sources for both periods of selection. The table also shows the p-values of the tests that investigated whether setting the weights equal to zero, one at a time, yielded a smaller profit. The p-values were obtained with t-tests from 200 replicates and show that most information sources significantly contributed to the realized profit. However, the effect of the heterozygosity of QTL 2 was not significantly different from zero when a Bonferroni correction was carried out to account for multiple testing. This was because the QTL had a low allele frequency throughout the whole breeding program.

During directional as well as stabilizing selection, a positive weight was given to the allele content $X_{i1}$ of QTL 1 and a negative weight was given to the allele content $X_{i2}$ of QTL 2. Hence, throughout the breeding program, the 1-allele of QTL 1 was driven to fixation, while the 1-allele of QTL 2 was eliminated. This led to a smaller phenotypic variance compared to when both QTL continued to segregate in the population. Fixation of the QTL alleles was reached at about generation 7. Selection against the 1-allele of QTL 2 was primarily done during stabilizing selection. This counteracted the effect of selection on QTL 1 and thus helped stabilizing the trait mean at the optimum. The QTL heterozygosities were handled differently during directional and stabilizing selection. During directional selection, the positive weight placed on the QTL heterozygosities favored animals with high Mendelian sampling variances for breeding, which led to an increase of the genetic variance in subsequent generations. The situation was reversed for stabilizing selection. The negative weight placed on the heterozygosities of QTL 1 and 2 favored homozygous animals for breeding since these animals had more copies of the favorable allele than heterozygous animals. The negative weight placed on $\frac{1}{2}(\text{EBV}_{i}-m_{s_i})^2$ during stabilizing selection favored the use of breeding animals with trait values from the center of the distribution. The resulting Bulmer effect led to a reduction of the genetic variance in subsequent generations.

**Fig. 2:**
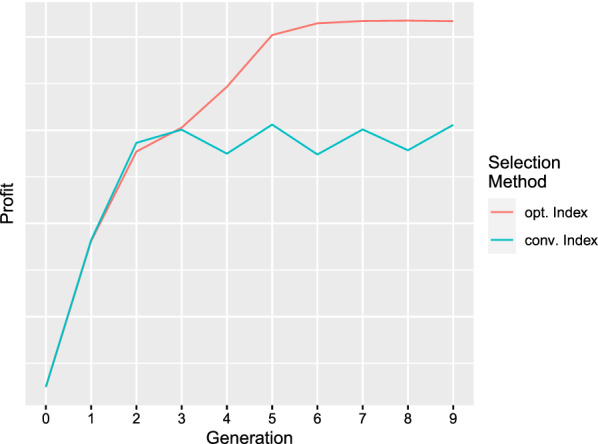
The profit of the simulated population in generations 0 to 9 is shown for two selection indices. The conventional index includes only the animal’s EBV, while the optimum index also includes the frequencies of major QTL, the QTL heterozygosities, and the squared EBV. The optimum index maximizes the average profit in generations 1 to 9

**Fig. 3:**
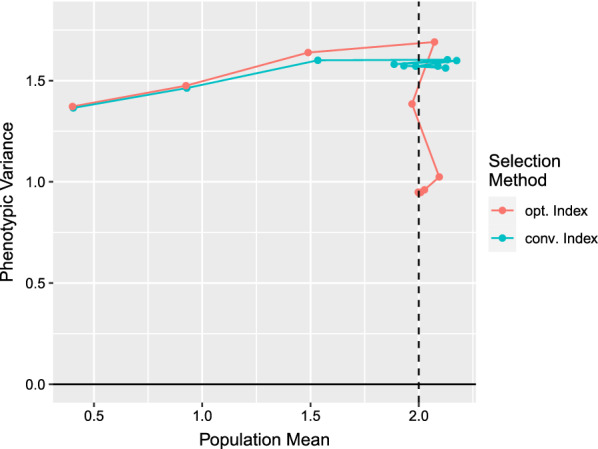
Change in population mean and phenotypic variance during the simulated breeding program for the conventional selection index and the optimum selection index. The parameter of generation 0 is shown by the point on the left-hand side. The conventional index oscillates around the optimum and cannot achieve any further genetic progress once the trait mean has reached the optimum. The optimum index achieves further progress by driving the major QTL to fixation, which reduces the phenotypic variance, and thus causes the phenotypes to be closer to the optimum

The optimum selection index was compared with a conventional selection index that included only the animal’s EBV. Figure 2 shows the profit of the population in generations 0 to 9 for the two selection indices. The optimum selection index led to additional gain in profit after the population mean has reached the optimum, while the conventional selection index achieved no further progress and caused, instead, the profit to oscillate. The parametric plot in Fig. 3 shows the change in population mean and the change in phenotypic variance during the course of the breeding program. The population in generation 0 is shown on the left-hand side. The conventional selection index caused the population mean to oscillate at the optimum. The optimum index achieved further progress by driving the major QTL to fixation, which reduced the phenotypic variance and thus caused the phenotypes to be closer to the optimum. The optimum index did not cause oscillation of the trait mean due to the restrictions imposed on $b_1$ and $b_2$.

In this example, it was not mandatory to use EBV. When the selection index was computed from the animal’s standardized phenotype rather than EBV, the time until the maximum profit was reached increased by only one generation (not shown).

**Table 2:** Optimum weights in the selection index for the simulated population, and p-values of the tests of whether setting the weights equal to zero would provide lower profit

Information source	Index coef.	Directional selection	Stabilizing selection	p-value
$\text{EBV}_{i}$	$b_1$	0.26	− 0.26 to 0.26	$4.4\cdot 10^{-20}$
$\text{EBV}_{i}^2$	$b_2$	− 0.06	− 0.26 to 0	$1.3\cdot 10^{-13}$
$\text{X}_{i1}$	$b_3$	0.46	0.08	$1.2\cdot 10^{-13}$
$\text{X}_{i2}$	$b_4$	− 0.04	− 0.42	$1.0\cdot 10^{-08}$
$\text{H}_{i1}$	$b_5$	0.18	− 0.22	$3.2\cdot 10^{-04}$
$\text{H}_{i2}$	$b_6$	0.00	− 0.02	0.02

The p-values are obtained with t-tests from 200 replicates

## Discussion

The selection index theory developed in this paper generalizes the traditional selection index theory to include stabilizing selection. The simple genetic model underlying traditional selection index theory is replaced by a general framework that can be applied to a variety of trait architectures. The theory was applied to various additive genetic models and estimation of optimum index weights was demonstrated by a simulation study (Fig. 3).

A selection index is an estimator of an animal’s aggregate genotype, which measures the expected increase in the breed’s profit in future generations that results from using the animal for breeding. The main part of the paper revealed the structure of the aggregate genotype and the genetic information on the selection candidate that influences its value. The structure of the aggregate genotype was elaborated for the important special case of a distance-based total merit function.

The definition of the partial merit functions by Eq. (4) excludes, at first glance, directional selection with a linear total merit function. Directional selection, is, however, included as a limiting case. The corresponding equations are obtained by setting $\chi _{\text{m}}^k(\tilde{\varvec{\upxi }}_n)=\pm 1$ in Eq. (7), and $\chi _{\text{v}}^k(\tilde{\varvec{\upxi }}_n)=0$ in Eq. (8).

When traits are under stabilizing selection, breeders will likely make compensatory matings. The models considered in this paper and the simulation study, however, assumed random mating. The application of selection indices that were derived under the assumption of a random mating population to non-random mating populations may require fine-tuning of the index weights to get optimum results. In addition, genetic drift and changes in breeding objectives over time may require updating the index weights after a few generations.

The developed framework can be extended to non-random mating populations by letting function $\varvec{\uptheta}_1$ not depend on the *N*-dimensional contribution vector $\bf{c}$ but on the mating list. A mating list is a $N\times N$-matrix $\varvec{L}$, where $L_{ij}$ equals the expected proportion of animals from the next generation that have animal *i* as the sire and animal *j* as the dam. The corresponding optimization problem then consists of finding the most suitable mating partner for a given animal that should be used to maximize the increase of the breed’s profit in future generations.

While traditional selection index theory provides an index that depends only on the breeding values, the optimum selection index depends also on the squared breeding values, on the allele contents of large QTL, on their heterozygosities, on the inbreeding coefficient of the animal, and on the kinship of the animal with the population. For traits under directional selection, the focus of the selection index is on improving the breeding values, while for traits under stabilizing selection, the focus is on the fixation of QTL with large effects. Fixation of QTL alleles reduces the phenotypic variance of the trait, which causes the phenotypes to be closer to the optimun and thereby increases the profit of the population. The purpose of the index weight on the kinship is to favor outcross animals for breeding when the trait is far away from the optimum, which tends to increase the additive variance in later generations, and can thus accelerate future selection response. Once the trait means have approached their optima, animals with high kinships might be favored for breeding to decrease the phenotypic variances. In this case, an appropriate weight on the kinship could only be derived from a model that accounts for dominance and inbreeding depression. In addition, fixation of large QTL can lead to local inbreeding and inbreeding depression. The models considered in this paper are purely additive, so they cannot capture these effects.

The simulation study revealed that benefit can be obtained from an optimum selection index. The simulation study also demonstrated that unlike the conventional selection index, the proposed selection index does not cause the population mean to oscillate at the optimum.

In dog breeding, breeding progress by reducing the genetic variances has traditionally been achieved by inbreeding. Inbreeding, however, is not an advisable breeding method because it can cause inbreeding depression. An alternative to inbreeding is to drive only the alleles of major QTL to fixation. The generalized selection index achieves this by putting appropriate weights on the allele contents, while continued stabilizing selection for the trait of interest ensures that the trait mean remains at the optimum. If this strategy is followed, then animals carrying the opposite alleles should be chosen for cryo-conservation.

The optimum selection index weights can either be computed in a simulation study, as demonstrated in the example, or they could be directly computed by the formulas that were derived in this paper. In the latter case, the selection index would be calculated as the index that has the highest correlation with the aggregate genotype defined in Eq. (1). This equation shows that the aggregate genotype depends on the breeding policy $\varvec\uppi$ that will be used in later generations. Thus, Eq. (1) is an iterative equation that provides the selection index for generation $n-1$ when the indices for generations $n,n+1,...$ are known. This implies that the following algorithm can be used to compute the optimal selection index weights. The algorithm starts with a putative state ${\varvec{\uptheta}}_n'$ of the population that is close to the desired final state $\breve{\varvec{\uptheta}}$, and determines an admissible vector ${\varvec{\uptheta}}_{n-1}'$ that is closer to the current state ${\varvec{\uptheta}}_0$ of the population. This involves computation of the aggregate genotype function $T_{{\varvec{\uptheta}}_{n-1}}({\bf{u}}_i)$ for generation $n-1$. This is repeated until ${\varvec{\uptheta}}_0'$ is reached. If ${\varvec{\uptheta}}_0'$ deviates from ${\varvec{\uptheta}}_0$, then the procedure is repeated with better guesses for *n* and ${\varvec{\uptheta}}_n'$. However, this naive procedure might not be efficient and further research is needed to develop and implement appropriate algorithms.

This paper modeled a breeding program as a dynamical system and the mapping ${\varvec\Gamma }_{\varvec\uppi}:\Theta \rightarrow \Theta$ determined the evolution of the system over time. For the optimum breeding policy, the desired final state $\breve{\varvec{\uptheta}}\in \Theta$ of the breeding program is likely the maximum of the profit function $\tilde{\phi }({\varvec{\uptheta}})=\phi ( \varvec\upxi (\varvec\uptheta))$, and simultaneously an attractor of the system. An attractor of a dynamical system is a set of states toward which the system tends to evolve for a wide range of starting conditions. The example showed that the desired final state is in general not uniquely defined. In the example, fixation of the alternative allele of QTL 1 and elimination of the alternative allele of QTL 2 would result in the same profit as elimination of the alternative allele of QTL 1 and fixation of the alternative allele of QTL 2. The path ${\varvec{\uptheta}}_1,{\varvec{\uptheta}}_2,...$ of the population through the state space maximizes

$$\begin{aligned} f({\varvec{\uptheta}}_1,{\varvec{\uptheta}}_2,...) = \sum \limits _{n=1}^\infty \zeta _n \tilde{\phi }({\varvec{\uptheta}}_n) \end{aligned}$$

under the condition that an aggregate genotype function $T_{{\varvec{\uptheta}}_n}$ exists for each state ${\varvec\uptheta}_n$ that can be used to bring the population into the subsequent state ${\varvec\uptheta}_{n+1}$. Such discrete-time dynamic optimization problems are often solved with optimum control theory or dynamic programming [25, 26].

**Fig. 4:**
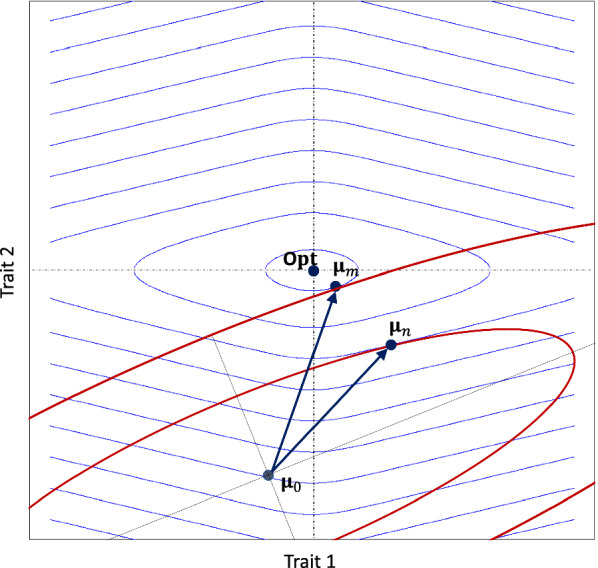
Illustration of the method of Moav and Hill [12] for finding the optimum selection index when genetic gain after *n* or $m>n$ generations is to be maximized. The level sets of the profit function from Eq. (5) are colored blue. The red ellipses are the response curves for *n* and *m* generations of selection. The traits are genetically correlated. Trait 1 has weight 0.2, a high heritability, and is close to the optimum. Trait 2 has weight 0.8, a lower heritability and is distant from the optimum

Under certain assumptions, the optimization problem can be solved with graphical methods. Suppose that genetic gain until the end of the planning horizon of the breeding program should be maximized. For a simple genetic model with constant genetic variances and covariances, ${\varvec\uptheta}_n={\varvec\upxi }_n={{\varvec\upmu }}_n$ can be assumed. Goddard [4] argued that the optimum approach in this case is the method of Moav and Hill [12], which is illustrated in Fig. 4. The goal is to achieve maximum profit after *n* generations of selection. The response curve, defined as the set of all population means that could be achieved until the end of the planning horizon is an ellipsoid with center ${{\varvec\upmu }}_0$. The point ${{\varvec\upmu }}_{n}$ on that ellipsoid with maximum profit after *n* generations of selection has the property that the level set for the profit function at this point is tangential to the response curve. The straight line from the current population mean ${{\varvec\upmu }}_0$ to the envisaged population mean ${{\varvec\upmu }}_{n}$ in generation *n* defines the optimum linear index that should be used.

The behavior of an optimum selection index is, in some cases, counter-intuitive. When the traits are correlated, and some traits are under directional selection and others are close to their optima, then the optimum selection index does not necessarily cause the latter traits to converge towards the optima because the negative profit arising from being away from the optimum could be offset by the greater responses of other traits [27]. This is also shown in Fig. 4. Moreover, Meuwissen and Goddard [28] pointed out that there can be non-linear relationships between production traits and fitness traits, in which case genetic correlations could change over time in an unpredictable manner, and that genetic drift in small populations may require a frequent updating of the index weights.

In the future, the selection index theory derived in this paper could be applied to an underlying genetic model that incorporates non-additive genetic effects, or a genetic heterogeneity of the environmental variance. Selection for reduced environmental variance could facilitate selection for uniformity and resilience [29].

Although the optimum selection index was derived under the assumption of truncation selection, in practice, the index can also be used for optimum contribution selection (OCS) [30–32]. In that case, kinship would be considered twice: first, as a constraint in OCS and, second, the selection index could prioritize outcross animals for breeding by placing a negative index weight on their kinship with the population. The developed method is, of course, most relevant to breeding program designs that meet the assumptions under which it was derived. These are breeding programs that implement truncation selection.

The selection index does not determine the optimum male for a given female. Mating decisions should be based primarily on the combining abilities between males and females. The combining ability of a male with a female predicts the total merit of their offspring. A recommended approach for mate allocation is to choose the male *i* that has the highest combining ability $C_{ij}$ with the female *j* that is to be mated, provided that the expected inbreeding coefficient of the offspring is sufficiently low, the female does not share any recessive deleterious alleles with the male, and the number of offspring of the male does not exceed the maximum foreseen number. Choosing the mating partner with the highest combining ability is also known as corrective mating [33]. This type of mate allocation can cause the progeny to have a higher expected total merit than the average total merit of the parents [34]. In some practical cases involving more comprehensive models, the combining ability for a mating depends on the other matings being made. For example, a dog breeding program could have a split objective that envisages trait bimodality for the trait activity level. Dogs with high activity level could be sold as working dogs, and the others as pet dogs. In this case, the value of a mating that generates progeny in the upper part of the distribution depends on whether that part of the distribution volume has already been met by other matings. In such cases a portfolio analysis is required, in which the impact of all matings to be made is evaluated simultaneously [35]. In a population without strong population structure, a reasonable superiority of mate allocation over random mating can, however, only be achieved for a trait with moderately high heritability that has a population mean close to the optimum, and if the total merit function has a peak at the optimum for that trait. This could be the case for many conformation traits. However, the extra gain from mate allocation is negligible for traits with low heritabilities, for traits under directional selection, and for total merit functions with an extended flat surface area around the optimum [36]. Implementing the proposed strategy for mate allocation requires the computation of combining abilities to be made feasible for breeders. This can be facilitated by an internet platform that provides the information. An alternative is a central mate allocation procedure that determines the complete set of mating pairs for a given generation that maximizes the average combining ability of the parents [37].

## Conclusion

In animal breeding, an increasing number of traits is under stabilizing selection, which is a situation in which traditional index selection cannot achieve further progress for those traits. To address this, selection index theory was derived that is applicable to both directional and stabilizing selection. For traits that are under directional selection, the index focuses on improving the breeding values, while for traits under stabilizing selection, the index focuses on fixation of QTL alleles. For traits under stabilizing selection, additional progress can be achieved by allocating mates such that the expected total merit of the offspring is maximized.

## Supplementary information

**Additional file 1.** Proofs of the formulas presented in the paper. The document has the classical structure of mathematical publicationsand contains definitions, theorems and proofs.
