# Supplementary material for: Selection index theory for populations under directional and stabilizing selection
Source: Genet Sel Evol. 2023 Feb 3;55:10. doi: 10.1186/s12711-023-00776-4 (PMC9898986; doi:10.1186/s12711-023-00776-4)
Supplement: Supplementary file 1 — Additional file 1. Proofs of the formulas presented in the paper. The document has the classical structure of mathematical publicationsand contains definitions, theorems and proofs. [file 12711_2023_776_MOESM1_ESM.pdf]

Proofs of the Paper:

Selection Index Theory for Populations under Directional and Stabilizing Selection

Robin Wellmann

September 19, 2022

Contents

1 Mathematical Background **3**

2 Selection Index Theory **5**

2.1 The General Case 5

2.2 Combination of Breeding Objectives 12

2.3 Distance-Based Merit Functions 14

3 Quantitative Genetic Theory **19**

3.1 Combining Ability 19

3.2 Simplistic Genetic Model 22

3.3 General Additive Model 24

3.4 QTL-based Additive Model with Polygenic Term 29

1 Mathematical Background

Definition 1. For $\mu \in \mathbb{R}$ and $\sigma > 0$ let

$$\psi(\mu, \sigma) = \sigma\sqrt{\frac{2}{\pi}}\exp\left(\frac{-\mu^2}{2\sigma^2}\right) + \mu\left(1 - 2\Phi\left(\frac{-\mu}{\sigma}\right)\right), \qquad (1)$$

where Φ is the cumulative distribution function of the standard normal distribution.

Lemma 1. For all $\mu \in \mathbb{R}$ and $\sigma > 0$ we have

$$\frac{\partial\psi}{\partial\mu}(\mu, \sigma) = 1 - 2\Phi\left(-\frac{\mu}{\sigma}\right),$$
$$\frac{\partial\psi}{\partial\sigma}(\mu, \sigma) = \sqrt{\frac{2}{\pi}}\exp\left(-\frac{\mu^2}{2\sigma^2}\right).$$

Proof:

We can write

$$\psi(\mu, \sigma) = \mu + \sigma\sqrt{\frac{2}{\pi}}\exp(g(\mu, \sigma)) - 2\mu(\Phi \circ h)(\mu, \sigma),$$

where the functions

$$g(\mu, \sigma) = \frac{-\mu^2}{2\sigma^2},$$
$$h(\mu, \sigma) = \frac{-\mu}{\sigma}$$

satisfy $g(\mu, \sigma) = \frac{-h(\mu,\sigma)^2}{2}$ and

$$\frac{\partial g}{\partial\mu}(\mu, \sigma) = -\frac{\mu}{\sigma^2},$$
$$\frac{\partial h}{\partial\mu}(\mu, \sigma) = -\frac{1}{\sigma},$$
$$\frac{\partial g}{\partial\sigma}(\mu, \sigma) = \frac{\mu^2}{\sigma^3},$$
$$\frac{\partial h}{\partial\sigma}(\mu, \sigma) = \frac{\mu}{\sigma^2}.$$

Moreover, $\frac{\partial\Phi}{\partial h}(h) = \frac{1}{\sqrt{2\pi}}\exp\left(-\frac{h^2}{2}\right)$. Hence,

$$
\begin{aligned}
\frac{\partial\psi}{\partial\mu}(\mu,\sigma) &= 1+\sigma\sqrt{\frac{2}{\pi}}\exp(g(\mu,\sigma))\,\frac{\partial g}{\partial\mu}(\mu,\sigma)-2\left(1(\Phi\circ h)(\mu,\sigma)+\mu\frac{\partial(\Phi\circ h)}{\partial\mu}(\mu,\sigma)\right),\\
&= 1+\sigma\sqrt{\frac{2}{\pi}}\exp(g(\mu,\sigma))\,\frac{\partial g}{\partial\mu}(\mu,\sigma)-2\Phi(h(\mu,\sigma))-2\mu\frac{\partial\Phi}{\partial h}(h(\mu,\sigma))\frac{\partial h}{\partial\mu}(\mu,\sigma)\\
&= 1-\sigma\sqrt{\frac{2}{\pi}}\exp(g(\mu,\sigma))\,\frac{\mu}{\sigma^2}-2\Phi(h(\mu,\sigma))+2\mu\frac{1}{\sqrt{2\pi}}\exp\left(-\frac{h(\mu,\sigma)^2}{2}\right)\frac{1}{\sigma}\\
&= 1-\frac{2\mu}{\sigma}\frac{1}{\sqrt{2\pi}}\exp(g(\mu,\sigma))-2\Phi(h(\mu,\sigma))+\frac{2\mu}{\sigma}\frac{1}{\sqrt{2\pi}}\exp\left(-\frac{h(\mu,\sigma)^2}{2}\right)\\
&= 1-\frac{2\mu}{\sigma}\frac{1}{\sqrt{2\pi}}\exp(g(\mu,\sigma))-2\Phi(h(\mu,\sigma))+\frac{2\mu}{\sigma}\frac{1}{\sqrt{2\pi}}\exp\left(g(\mu,\sigma)\right)\\
&= 1-2\Phi(h(\mu,\sigma))\\
&= 1-2\Phi\left(\frac{-\mu}{\sigma}\right)
\end{aligned}
$$

and

$$
\begin{aligned}
\frac{\partial\psi}{\partial\sigma}(\mu,\sigma) &= 0+\sqrt{\frac{2}{\pi}}\exp(g(\mu,\sigma))+\sigma\sqrt{\frac{2}{\pi}}\exp(g(\mu,\sigma))\,\frac{\partial g}{\partial\sigma}(\mu,\sigma)-2\mu\frac{\partial\Phi}{\partial h}(h(\mu,\sigma))\frac{\partial h}{\partial\sigma}(\mu,\sigma)\\
&= \sqrt{\frac{2}{\pi}}\exp(g(\mu,\sigma))+\sigma\sqrt{\frac{2}{\pi}}\exp(g(\mu,\sigma))\,\frac{\partial g}{\partial\sigma}(\mu,\sigma)-2\mu\frac{1}{\sqrt{2\pi}}\exp\left(-\frac{h(\mu,\sigma)^2}{2}\right)\frac{\partial h}{\partial\sigma}(\mu,\sigma)\\
&= \sqrt{\frac{2}{\pi}}\exp(g(\mu,\sigma))+\sigma\sqrt{\frac{2}{\pi}}\exp(g(\mu,\sigma))\,\frac{\partial g}{\partial\sigma}(\mu,\sigma)-\mu\sqrt{\frac{2}{\pi}}\exp\left(g(\mu,\sigma)\right)\frac{\partial h}{\partial\sigma}(\mu,\sigma)\\
&= \sqrt{\frac{2}{\pi}}\exp(g(\mu,\sigma))\left(1+\sigma\frac{\partial g}{\partial\sigma}(\mu,\sigma)-\mu\frac{\partial h}{\partial\sigma}(\mu,\sigma)\right)\\
&= \sqrt{\frac{2}{\pi}}\exp(g(\mu,\sigma))\left(1+\sigma\frac{\mu^2}{\sigma^3}-\mu\frac{\mu}{\sigma^2}\right)\\
&= \sqrt{\frac{2}{\pi}}\exp(g(\mu,\sigma))\\
&= \sqrt{\frac{2}{\pi}}\exp\left(-\frac{\mu^2}{2\sigma^2}\right)
\end{aligned}
$$

□

2 Selection Index Theory

It is assumed throughout the paper that the population is diploid and random mating with two sexes.

2.1 The General Case

Definition 2. A total merit function is a function $\mathrm{TM} : \mathbb{R}^K \to \mathbb{R}$, where K is the number of traits. The total merit of individual i with phenotype vector $y_i \in \mathbb{R}^K$ is thus denoted as $\mathrm{TM}(y_i)$.

Definition 3. Let Ξ denote the parameter space of the phenotypic distribution, and let $f(y|\xi)$ denote its density. Suppose that the function $\xi \mapsto f(y|\xi)$ is differentiable for all $y \in \mathbb{R}^K$. The profit $\phi(\xi)$ of a population with parameter $\xi \in \Xi$ is given by the function

$$\phi : \Xi \to \mathbb{R}, \phi(\xi) \;=\; E_\xi(\mathrm{TM}(Y)) \;=\; \int \mathrm{TM}(y) f(y|\xi)\, dy,$$

where the random K-vector Y contains the phenotype of an individual that is randomly chosen from the population.

Definition 4. For a population with two sexes and N selection candidates, the set

$$\mathcal{C} = \left\{ c \in \mathbb{R}^N_{\geq 0} : \sum_{i:i \text{ male}} c_i = 0.5, \quad \text{and} \quad \sum_{i:i \text{ female}} c_i = 0.5 \right\}$$

is called the set of genetic contribution vectors.

Lemma 2. In order to simplify notations, we denote with $\mathrm{s}_i \in \{\mathrm{m}, \mathrm{f}\}$ the sex of individual i and with $\bar{\mathrm{s}}_i$ the opposite sex. Each contribution vector $c \in \mathcal{C}$ has a representation

$$c \;=\; c_{\mathrm{m}} + c_{\mathrm{f}} \;=\; c_{\mathrm{s}_i} + c_{\bar{\mathrm{s}}_i},$$

where the vector c_{m} with male contributions equals zero for females, and the vector c_{f} with female contributions equals zero for males.

Proof: This is clear. □

Definition 5. A population is called an idealized population with state space Θ, if the following conditions are satisfied:

- The function $\theta_1 : \mathcal{C} \to \Theta$ that provides the expected state of the population in generation 1 as a function of the vector $c \in \mathcal{C}$ with genetic contributions is differentiable.
- There exists a differentiable function $\Gamma : \Theta \to \Theta$ that provides the state $\theta_{n+1} = \Gamma(\theta_n)$ of the population in each next generation.
- There exists a differentiable function $\xi : \Theta \to \Xi$ that extracts the parameter of the phenotypic distribution from the population's state.

Note that the mapping Γ would be approximate for finite, real existing populations.

Definition 6. The mapping $\Gamma = \Gamma_\pi$ from Definition 5 and the state $\theta_n = \theta_n^\pi$ of the population in generation n are determined by the breeding policy π of the population. The set of breeding policies is denoted as Π. Each breeding policy $\pi = (T_\theta, \mathrm{tr}_\theta)_{\theta \in \Theta}$ provides for a given state θ of the population a point of truncation tr_θ, and a function

$$T_\theta : \mathcal{U} \to \mathbb{R}, u_i \mapsto T_\theta(u_i),$$

which is called the aggregate genotype function. Thereby, $\mathcal{U}$ denotes the set of information sources. A vector $u_i \in \mathcal{U}$ contains the unknown true genetic information on individual i that could affect selection decisions, and $\hat{u}_i$ is an estimate of u_i.

The aggregate genotype function T_θ is used for calculating the selection index I_i of each individual i. An individual is used for breeding if $I_i > \mathrm{tr}_\theta$.

Definition 7. The expected profit of a breed after $n \geq 1$ generations of selection by following breeding policy π is defined as

$$\phi_n(\pi) \quad = \quad E_\pi(\mathrm{TM}(Y_n)|\theta_0, u, \hat{u}),$$

where

Y_n is the random K-vector with phenotypic values of an individual that is chosen from generation n,

θ_0 is the current state of the population,

u is the $N \times L$ matrix with the unknown true genetic values of the N selection candidates,

$\hat{u}$ is the $N \times L$ matrix with estimated genetic values of the N selection candidates.

Definition 8. Let $\zeta = (\zeta_n)_{n\geq1}$ be a sequence with $\zeta_n \geq 0$ and $\sum_{n=1}^{\infty} \zeta_n = 1$. Then, ζ_n is called the importance placed on the profit of the breed in generation n, and the expected future profit of the population is defined as

$$f(\pi) \quad = \quad \sum_{n=1}^{\infty} \zeta_n \phi_n(\pi).$$

Lemma 3. For an idealized population, the expected future profit of the population equals

$$f(\pi) \quad = \quad \tilde{f}_\pi(c_\pi),$$

where $c_\pi \in \mathcal{C}$ is the vector with genetic contributions of the selection candidates, and

$$\tilde{f}_\pi(c) \quad = \quad \sum_{n=1}^{\infty} \zeta_n \phi(\xi(\theta_n^\pi(c)).$$

Thereby,

$$\theta_n^\pi : \mathcal{C} \to \Theta, \;\; \theta_n^\pi(c) = \Gamma_\pi^{n-1}(\theta_1(c))$$

provides the state of the population in generation $n \geq 1$ as a function of the genetic contributions c_i of the selection candidates. Note that θ_1 depends, in fact, also on the matrix $u \in \mathcal{U}^N$ with true genetic values of the selection candidates, and on the current state θ_0 of the population, while c_π depends on the matrix $\hat{u} \in \mathcal{U}^N$ with estimated genetic values. The parameters u and $\hat{u}$, the vector θ_0, and the superscript π are often omitted in the following to keep the formulas readable.

Proof:

$$\begin{aligned}
f(\pi) &\overset{\text{Def.8}}{=} \sum_{n=1}^{\infty} \zeta_n \phi_n(\pi) \\
&\overset{\text{Def.7}}{=} \sum_{n=1}^{\infty} \zeta_n E_\pi(\mathrm{TM}(Y_n)|\theta_0, u, \hat{u}) \\
&= \sum_{n=1}^{\infty} \zeta_n E_{\xi(\theta_n^\pi(c_\pi))}(\mathrm{TM}(Y_n)) \\
&= \sum_{n=1}^{\infty} \zeta_n \phi(\xi(\theta_n^\pi(c_\pi))
\end{aligned}$$

□

Definition 9. The vector $\tilde{\boldsymbol{c}} \in \mathcal{C}$ with default contributions is a vector for which $\tilde{\theta}_1 = \boldsymbol{\theta}_1(\tilde{c})$ is approximately the expected state of the population in the next generation.

Definition 10. For an idealized population, the aggregate genotype of individual i is defined as

$$T_i \quad = \quad \lim_{\lambda \to 0} \frac{\tilde{f}_\pi\left(\tilde{c} + \lambda \tilde{e}_i\right) - \tilde{f}_\pi(\tilde{c})}{\lambda},$$

where $\tilde{c} \in \mathcal{C}$ is the vector with default contributions, $\tilde{e}_i = e_i - 2\tilde{c}_{\mathrm{s}_i}$, and e_i denotes the standard unit vector.

Note that the aggregate genotype T_i measures the increase of the breed's future profit that results from using individual i for breeding.

Lemma 4. For an idealized population, the aggregate genotype satisfies

$$T_i \quad = \quad \frac{\partial \tilde{f}_\pi}{\partial c_i}(\tilde{c}) - \nu_{\mathrm{s}_i}$$

with $\nu_{\mathrm{s}_i} = 2\tilde{c}_{s_i}^\top \nabla \tilde{f}_\pi|_{\tilde{c}}$, where $\nabla \tilde{f}_\pi|_{\tilde{c}}$ is the gradient of $\tilde{f}_\pi$ at $\tilde{c}$.

Proof:

Function $\tilde{f}_\pi$ is differentiable, so the limit does not change, when $\tilde{f}_\pi$ is replaced by the first order Taylor approximation

$$\tilde{f}_\pi\left(\tilde{c} + \lambda \tilde{e}_i\right) \quad \approx \quad \tilde{f}_\pi(\tilde{c}) + (\lambda \tilde{e}_i)^\top \nabla \tilde{f}_\pi|_{\tilde{c}}.$$

Thus,

$$\begin{aligned}
T_i &= \lim_{\lambda \to 0} \frac{\tilde{f}_\pi\left(\tilde{c} + \lambda \tilde{e}_i\right) - \tilde{f}_\pi(\tilde{c})}{\lambda} \\
&= \lim_{\lambda \to 0} \frac{\tilde{f}_\pi(\tilde{c}) + (\lambda \tilde{e}_i)^\top \nabla \tilde{f}_\pi|_{\tilde{c}} - \tilde{f}_\pi(\tilde{c})}{\lambda} \\
&= \lim_{\lambda \to 0} \frac{\lambda \tilde{e}_i^\top \nabla \tilde{f}_\pi|_{\tilde{c}}}{\lambda} \\
&= \tilde{e}_i^\top \nabla \tilde{f}_\pi|_{\tilde{c}} \\
&= e_i^\top \nabla \tilde{f}_\pi|_{\tilde{c}} - 2\tilde{c}_{s_i}^\top \nabla \tilde{f}_\pi|_{\tilde{c}} \\
&= \frac{\partial \tilde{f}_\pi}{\partial c_i}(\tilde{c}) - \nu_{\mathrm{s}_i}.
\end{aligned}$$

□

Theorem 1. For an idealized population, the aggregate genotype satisfies

$$T_i = v(\tilde{\theta}_1)^\top u_i - \nu_{\mathrm{s}_i}$$

where

$$u_i = \frac{\partial \theta_1}{\partial c_i}(\tilde{c})$$

is called the vector with true genetic values of individual i, and

$$v(\tilde{\theta}_1)^\top = \sum_{n=1}^{\infty} \zeta_n \frac{\partial \phi}{\partial \xi_n}(\tilde{\xi}_n) \cdot \frac{\partial \xi_n}{\partial \theta_1}(\tilde{\theta}_1)$$

is called the vector with economic weights. Thereby, the derivative of a vector by a vector is the Jacobi matrix with partial derivatives, $\xi_n(\theta_1) = \xi(\Gamma_\pi^{n-1}(\theta_1))$, $\tilde{\xi}_n = \xi_n(\tilde{\theta}_1)$, and $\tilde{\theta}_1 = \theta_1(\tilde{c})$.

Proof:

$$T_i \overset{\text{Lem.4}}{=} \frac{\partial}{\partial c_i} \tilde{f}_\pi(\tilde{c}) - \nu_{\mathrm{s}_i},$$

where

$$\begin{aligned}
\frac{\partial}{\partial c_i} \tilde{f}_\pi(\tilde{c}) &\overset{\text{Lem.3}}{=} \frac{\partial}{\partial c_i} \sum_{n=1}^{\infty} \zeta_n \phi(\xi_n^\pi(\tilde{c})) \\
&= \sum_{n=1}^{\infty} \zeta_n \frac{\partial}{\partial c_i} \phi(\xi_n^\pi(\tilde{c})) \\
&= \sum_{n=1}^{\infty} \zeta_n \frac{\partial \phi}{\partial \xi_n}(\tilde{\xi}_n) \frac{\partial \xi_n}{\partial \theta_1}(\tilde{\theta}_1) \frac{\partial \theta_1}{\partial c_i}(\tilde{c}) \\
&= v(\tilde{\theta}_1)^\top u_i.
\end{aligned}$$

□

Theorem 2. Let $v(\tilde{\theta}_1)$ be the vector with economic weights, let u_i be the L-vector with true genetic values of animal i and let $\hat{u}_i$ be an estimate thereof. Suppose that either animal i is chosen at random from a specific class of animals, or that u_i and $\hat{u}_i$ are random for other reasons. Let

$$P_i = \Sigma_{\hat{u}_i\hat{u}_i} = \mathrm{Cov}(\hat{u}_i), \text{ and } G_i = \Sigma_{\hat{u}_i u_i} = \mathrm{Cov}(\hat{u}_i, u_i).$$

Then, the function

$$r(b_i) \;=\; \mathrm{corr}(b_i^\top \hat{u}_i, v(\tilde{\theta}_1)^\top u_i)$$

has its maximum at

$$b_i \;=\; P_i^{-1} G_i \, v(\tilde{\theta}_1).$$

Proof:

The proof is analogous to the corresponding proof in traditional index selection. Let

$$P_i = \Sigma_{\hat{u}_i\hat{u}_i} = \mathrm{Cov}(\hat{u}_i), \text{ and } G_i = \Sigma_{\hat{u}_i u_i} = \mathrm{Cov}(\hat{u}_i, u_i).$$

The function

$$r(b_i) \;=\; \mathrm{corr}(b_i^\top \hat{u}_i, v(\tilde{\theta}_1)^\top u_i) = \frac{\mathrm{Cov}(b_i^\top \hat{u}_i, v(\tilde{\theta}_1)^\top u_i)}{\sqrt{\mathrm{Var}(b_i^\top \hat{u}_i)}\sqrt{\mathrm{Var}(v(\tilde{\theta}_1)^\top u_i)}}$$

has its maximum at the same position as function

$$\tilde{r}(b_i) \;=\; \frac{\mathrm{Cov}(b_i^\top \hat{u}_i, v(\tilde{\theta}_1)^\top u_i)}{\sqrt{\mathrm{Var}(b_i^\top \hat{u}_i)}}$$

We have

$$\tilde{r}(b_i) \;=\; \frac{b_i^\top \mathrm{Cov}(\hat{u}_i, u_i) v(\tilde{\theta}_1)}{\sqrt{b_i^\top \mathrm{Cov}(\hat{u}_i) b_i}} \;=\; \frac{b_i^\top G_i v(\tilde{\theta}_1)}{\sqrt{b_i^\top P_i b_i}} \;=\; \frac{\psi(b_i)}{\varphi(b_i)}$$

with $\psi(b_i) = b_i^\top G_i v(\tilde{\theta}_1)$, and $\varphi(b_i) = \sqrt{b_i^\top P_i b_i}$. Moreover,

$$\begin{aligned}
\nabla\psi(b_i) &= G_i v(\tilde{\theta}_1) \\
\nabla\varphi(b_i) &= \frac{1}{2}(b_i^\top P_i b_i)^{-\frac{1}{2}} 2 P_i b_i \\
&= \frac{1}{\sqrt{b_i^\top P_i b_i}} P_i b_i.
\end{aligned}$$

Thus,

$$\nabla\tilde{r}(b_i) \;=\; \frac{\varphi(b_i)\nabla\psi(b_i) - \psi(b_i)\nabla\varphi(b_i)}{\varphi(b_i)^2},$$

which equals zero if

$$\begin{aligned}
\sqrt{b_i^\top P_i b_i}\, G_i v(\tilde{\theta}_1) &= \varphi(b_i)\nabla\psi(b_i) \\
&= \psi(b_i)\nabla\varphi(b_i) \\
&= b_i^\top G_i v(\tilde{\theta}_1)\frac{1}{\sqrt{b_i^\top P b_i}} P b_i.
\end{aligned}$$

Consequently,

$$P b_i = \frac{b_i^\top P_i b_i}{b_i^\top G_i v(\tilde{\theta}_1)} G_i v(\tilde{\theta}_1).$$

Thus,

$$b_i = \frac{b_i^\top P_i b_i}{b_i^\top G_i v(\tilde{\theta}_1)} P_i^{-1} G_i v(\tilde{\theta}_1).$$

The equation holds for

$$b_i = \pm P_i^{-1} G_i v(\tilde{\theta}_1),$$

whereby the maximum is obtained for

$$b_i = P_i^{-1} G_i v(\tilde{\theta}_1),$$

but also for λb_i with $\lambda > 0$.

□

2.2 Combination of Breeding Objectives

Definition 11. We call a total merit function "general", if the function combines several partial merit functions into a single one. That is, a total merit function is general if there is a set $\mathcal{O}$ of breeding objectives, and a partial merit function $\mathrm{PM}_o : \mathbb{R}^K \to \mathbb{R}$ for each breeding objective o, such that

$$\mathrm{TM}(y) = \sum_{o \in \mathcal{O}} w_o \mathrm{PM}_o(y)$$

for all $y \in \mathbb{R}^K$.

Lemma 5. For a general total merit function, the profit function is

$$\phi(\xi) = \sum_{o \in \mathcal{O}} w_o \phi_o(\xi),$$

where

$$\phi_o(\xi) = E_\xi(\mathrm{PM}_o(Y))$$

is called the breed's profit with respect to breeding objective o, and Y is the K-vector with phenotypic values of an individual that is randomly from a population with parameter ξ.

Proof:

$$\begin{aligned}
\phi(\xi) &\overset{\text{Def.3}}{=} E_\xi(\mathrm{TM}(Y)) \\
&\overset{\text{Def.11}}{=} E_\xi\left(\sum_{o \in \mathcal{O}} w_o \mathrm{PM}_o(Y)\right) \\
&= \sum_{o \in \mathcal{O}} w_o E_\xi(\mathrm{PM}_o(Y)) \\
&= \sum_{o \in \mathcal{O}} w_o \phi_o(\xi),
\end{aligned}$$

□

Theorem 3. For an idealized population and a general total merit function, the vector with economic weights satisfies

$$v(\tilde{\theta}_1) = \sum_{o \in \mathcal{O}} w_o v_o(\tilde{\theta}_1),$$

where

$$v_o(\tilde{\theta}_1)^\top = \sum_{n=1}^{\infty} \zeta_n \frac{\partial \phi_o}{\partial \xi_n}(\tilde{\xi}_n) \cdot \frac{\partial \xi_n}{\partial \theta_1}(\tilde{\theta}_1)$$

is called the vector with economic weights for breeding objective o. Thereby, the derivative of a vector by a vector is the Jacobi matrix with partial derivatives, $\xi_n(\theta_1) = \xi(\Gamma_\pi^{n-1}(\theta_1))$, $\tilde{\xi}_n = \xi_n(\tilde{\theta}_1)$, and $\tilde{\theta}_1 = \theta_1(\tilde{c})$.

Proof:

$$\begin{aligned}
\frac{\partial \phi}{\partial \xi_n}(\tilde{\xi}_n) &\overset{\text{Lem.5}}{=} \frac{\partial}{\partial \xi_n} \sum_{o \in \mathcal{O}} w_o \phi_o(\tilde{\xi}_n) \\
&= \sum_{o \in \mathcal{O}} w_o \frac{\partial \phi_o}{\partial \xi_n}(\tilde{\xi}_n)
\end{aligned}$$

Thus,

$$\begin{aligned}
v(\tilde{\theta}_1)^\top &\overset{\text{Theo.1}}{=} \sum_{n=1}^{\infty} \zeta_n \frac{\partial \phi}{\partial \xi_n}(\tilde{\xi}_n) \cdot \frac{\partial \xi_n}{\partial \theta_1}(\tilde{\theta}_1) \\
&= \sum_{n=1}^{\infty} \zeta_n \sum_{o \in \mathcal{O}} w_o \frac{\partial \phi_o}{\partial \xi_n}(\tilde{\xi}_n) \cdot \frac{\partial \xi_n}{\partial \theta_1}(\tilde{\theta}_1) \\
&= \sum_{o \in \mathcal{O}} w_o \sum_{n=1}^{\infty} \zeta_n \frac{\partial \phi_o}{\partial \xi_n}(\tilde{\xi}_n) \cdot \frac{\partial \xi_n}{\partial \theta_1}(\tilde{\theta}_1) \\
&= \sum_{o \in \mathcal{O}} w_o \, v_o(\tilde{\theta}_1)^\top.
\end{aligned}$$

□

2.3 Distance-Based Merit Functions

Definition 12. We call a general total merit function "distance-based" and "piecewise linear", if all partial merit functions have the representation

$$\mathrm{PM}_o(y_i) \quad = \quad \tau_{\max} - \sum_{k=1}^{K} \omega_{ok} \left| y_{ik} - \mathrm{Opt}_{ok} \right|,$$

where ω_{ok} is the weight of trait k, Opt_{ok} is the optimum of trait k for breeding objective o, and $\tau_{\max}$ is an arbitrary constant.

Definition 13. The parameter space Ξ for a piecewise linear total merit function and normally distributed traits consists of all tuples $\xi = (\mu, \sigma_{\mathrm{P}})$ with $\mu \in \mathbb{R}^K$ and $\sigma_{\mathrm{P}} \in \mathbb{R}^K_{>0}$.

The parameter space could alternatively be defined as consisting of of all tuples $\xi = (\mu, \Sigma_{\mathrm{P}})$, where Σ_{P} is the phenotypic covariance matrix. The next lemma

shows that this is not needed because the profit of the population does not depend on the phenotypic correlations.

Lemma 6. Suppose that the total merit function is distance-based and piecewise linear, and that all traits are normally distributed. Then, the profit of the population for breeding objective o is

$$\phi_o(\xi) \quad = \quad \tau_{\max} - \sum_{k=1}^{K} \omega_{ok}\, \psi(\mu_k - \mathrm{Opt}_{ok}, \sigma_{\mathrm{P}k}).$$

Proof:

$$\begin{aligned}
\phi_o(\xi) &\stackrel{\text{Lem.5}}{=} E_\xi(\mathrm{PM}_o(Y)) \\
&\stackrel{\text{Def.12}}{=} \tau_{\max} - \sum_{k=1}^{K} \omega_{ok} E_\xi(|Y_k - \mathrm{Opt}_{ok}|) \\
&= \tau_{\max} - \sum_{k=1}^{K} \omega_{ok}\, \psi(\mu_k - \mathrm{Opt}_{ok}, \sigma_{\mathrm{P}k})
\end{aligned}$$

The last equality holds because $|Y_k - \mathrm{Opt}_{ok}|$ has a folded normal distribution.

□

Lemma 7. Suppose that the total merit function is distance-based and piecewise linear, and that all traits are normally distributed. Then, the profit of the population for breeding objective o has partial derivatives

$$\begin{aligned}
\frac{\partial \phi_o}{\partial \mu_k}(\xi) &= \omega_{ok}\, \chi_{\mathrm{m}}^k(\xi), \\
\frac{\partial \phi_o}{\partial \sigma_{\mathrm{P}k}}(\xi) &= \omega_{ok}\, \chi_{\mathrm{v}}^k(\xi),
\end{aligned}$$

with $\xi = (\mu, \sigma_{\mathrm{P}})$, and

$$\begin{aligned}
\chi_{\mathrm{m}}^k(\xi) &= 2\Phi\left(\frac{\mathrm{Opt}_{ok} - \mu_k}{\sigma_{\mathrm{P}k}}\right) - 1, \\
\chi_{\mathrm{v}}^k(\xi) &= -\sqrt{\frac{2}{\pi}} \exp\left(-\frac{(\mu_k - \mathrm{Opt}_{ok})^2}{2\sigma_{\mathrm{P}k}^2}\right).
\end{aligned}$$

Proof:

First equation: We have

$$\phi_o(\xi) \overset{\text{Lem.6}}{=} \tau_{\max} - \sum_{k=1}^{K} \omega_{ok}\psi(\mu_k - \mathrm{Opt}_{ok}, \sigma_{\mathrm{P}k}),$$

so

$$\begin{aligned}
\frac{\partial \phi_o}{\partial \mu_k}(\xi) &= -\omega_{ok}\frac{\partial \psi}{\partial \mu_k}(\mu_k - \mathrm{Opt}_{ok}, \sigma_{\mathrm{P}k}) \\
&\overset{\text{Lem.1}}{=} -\omega_{ok}\left(1 - 2\Phi\left(-\frac{\mu_k - \mathrm{Opt}_{ok}}{\sigma_{\mathrm{P}k}}\right)\right) \\
&= -\omega_{ok}\left(1 - 2\Phi\left(\frac{\mathrm{Opt}_{ok} - \mu_k}{\sigma_{\mathrm{P}k}}\right)\right) \\
&= \omega_{ok}\left(2\Phi\left(\frac{\mathrm{Opt}_{ok} - \mu_k}{\sigma_{\mathrm{P}k}}\right) - 1\right) \\
&= \omega_{ok}\ \chi_{\mathrm{m}}^{k}(\xi)
\end{aligned}$$

Second equation:

$$\begin{aligned}
\frac{\partial \phi_o}{\partial \sigma_{\mathrm{P}k}}(\xi) &= -\omega_{ok}\frac{\partial \psi}{\partial \sigma_{Pk}}(\mu_k - \mathrm{Opt}_{ok}, \sigma_{\mathrm{P}k}) \\
&\overset{\text{Lem.1}}{=} -\omega_{ok}\sqrt{\frac{2}{\pi}}\exp\left(-\frac{(\mu_k - \mathrm{Opt}_{ok})^2}{2\sigma_{\mathrm{P}k}^2}\right) \\
&= \omega_{ok}\ \chi_{\mathrm{v}}^{k}(\xi)
\end{aligned}$$

□

Theorem 4. Suppose that the total merit function is distance-based and piecewise linear, and that all traits are normally distributed. Then, the vector with economic weights is

$$v_o(\tilde{\theta}_1) \quad = \quad \sum_{k=1}^{K} \omega_{ok} v_k(\tilde{\theta}_1)$$

with

$$v_k(\tilde{\theta}_1) \quad = \quad v_k^{\mathrm{m}}(\tilde{\theta}_1) + v_k^{\mathrm{v}}(\tilde{\theta}_1),$$

and

$$\begin{aligned} v_k^{\mathrm{m}}(\tilde{\theta}_1)^\top &= \sum_{n=1}^{\infty} \zeta_n \, \chi_{\mathrm{m}}^k(\tilde{\xi}_n) \, \frac{\partial \mu_{nk}}{\partial \theta_1}(\tilde{\theta}_1), \\ v_k^{\mathrm{v}}(\tilde{\theta}_1)^\top &= \sum_{n=1}^{\infty} \zeta_n \, \chi_{\mathrm{v}}^k(\tilde{\xi}_n) \frac{\partial \sigma_{\mathrm{P}nk}}{\partial \theta_1}(\tilde{\theta}_1). \end{aligned}$$

Proof:

$$
\begin{aligned}
v_o(\tilde{\theta}_1)^\top &\overset{\text{Theo.3}}{=} \sum_{n=1}^{\infty} \zeta_n \, \frac{\partial \phi_o}{\partial \xi_n}(\tilde{\xi}_n) \cdot \frac{\partial \xi_n}{\partial \theta_1}(\tilde{\theta}_1) \\
&= \sum_{n=1}^{\infty} \zeta_n \, \frac{\partial \phi_o}{\partial \xi_n}(\tilde{\xi}_n) \cdot \frac{\partial \xi_n}{\partial \theta_n}(\tilde{\theta}_n) \cdot \frac{\partial \theta_n}{\partial \theta_1}(\tilde{\theta}_1) \\
&= \sum_{n=1}^{\infty} \zeta_n \, \left(\frac{\partial \phi_o}{\partial \mu_n}(\tilde{\xi}_n), \frac{\partial \phi_o}{\partial \sigma_{\mathrm{P}n}}(\tilde{\xi}_n) \right) \cdot \begin{pmatrix} \frac{\partial \mu_n}{\partial \theta_n}(\tilde{\theta}_n) \\ \frac{\partial \sigma_{\mathrm{P}n}}{\partial \theta_n}(\tilde{\theta}_n) \end{pmatrix} \cdot \frac{\partial \theta_n}{\partial \theta_1}(\tilde{\theta}_1) \\
&= \sum_{n=1}^{\infty} \zeta_n \, \left(\sum_{k=1}^{K} \frac{\partial \phi_o}{\partial \mu_{nk}}(\tilde{\xi}_n) \frac{\partial \mu_{nk}}{\partial \theta_n}(\tilde{\theta}_n) + \sum_{k=1}^{K} \frac{\partial \phi_o}{\partial \sigma_{\mathrm{P}nk}}(\tilde{\xi}_n) \frac{\partial \sigma_{\mathrm{P}nk}}{\partial \theta_n}(\tilde{\theta}_n) \right) \cdot \frac{\partial \theta_n}{\partial \theta_1}(\tilde{\theta}_1) \\
&= \sum_{n=1}^{\infty} \zeta_n \, \sum_{k=1}^{K} \frac{\partial \phi_o}{\partial \mu_{nk}}(\tilde{\xi}_n) \frac{\partial \mu_{nk}}{\partial \theta_n}(\tilde{\theta}_n) \cdot \frac{\partial \theta_n}{\partial \theta_1}(\tilde{\theta}_1) \\
&+ \sum_{n=1}^{\infty} \zeta_n \, \sum_{k=1}^{K} \frac{\partial \phi_o}{\partial \sigma_{\mathrm{P}nk}}(\tilde{\xi}_n) \frac{\partial \sigma_{\mathrm{P}nk}}{\partial \theta_n}(\tilde{\theta}_n) \cdot \frac{\partial \theta_n}{\partial \theta_1}(\tilde{\theta}_1) \\
&\overset{\text{Lem.7}}{=} \sum_{n=1}^{\infty} \zeta_n \, \sum_{k=1}^{K} \omega_{ok} \, \chi_{\mathrm{m}}^{k}(\tilde{\xi}_n) \frac{\partial \mu_{nk}}{\partial \theta_n}(\tilde{\theta}_n) \cdot \frac{\partial \theta_n}{\partial \theta_1}(\tilde{\theta}_1) \\
&+ \sum_{n=1}^{\infty} \zeta_n \, \sum_{k=1}^{K} \omega_{ok} \, \chi_{\mathrm{v}}^{k}(\tilde{\xi}_n) \frac{\partial \sigma_{\mathrm{P}nk}}{\partial \theta_n}(\tilde{\theta}_n) \cdot \frac{\partial \theta_n}{\partial \theta_1}(\tilde{\theta}_1) \\
&= \sum_{k=1}^{K} \omega_{ok} \sum_{n=1}^{\infty} \zeta_n \, \chi_{\mathrm{m}}^{k}(\tilde{\xi}_n) \frac{\partial \mu_{nk}}{\partial \theta_1}(\tilde{\theta}_1) \\
&+ \sum_{k=1}^{K} \omega_{ok} \sum_{n=1}^{\infty} \zeta_n \, \chi_{\mathrm{v}}^{k}(\tilde{\xi}_n) \frac{\partial \sigma_{\mathrm{P}nk}}{\partial \theta_1}(\tilde{\theta}_1) \\
&= \sum_{k=1}^{K} \omega_{ok} v_k^{\mathrm{m}}(\tilde{\theta}_1)^\top + \sum_{k=1}^{K} \omega_{ok} v_k^{\mathrm{v}}(\tilde{\theta}_1)^\top \\
&= \sum_{k=1}^{K} \omega_{ok} \left(v_k^{\mathrm{m}}(\tilde{\theta}_1) + v_k^{\mathrm{v}}(\tilde{\theta}_1) \right)^\top
\end{aligned}
$$

$\square$

3 Quantitative Genetic Theory

Definition 14. The following notations are used

TBV_{ik}: True breeding value of individual i for trait k

MT_{ik}: Mendelian sampling term for trait k for a haplotype that is transmitted by individual i to its offspring.

MV_{ik}: Mendelian sampling variance. It is the variance of the Mendelian sampling term of trait k that is submitted by individual i to its offspring.

X_{iq}: Allele content of individual i for QTL q. That is, $X_{iq} \in \{0, 1, 2\}$ is the number of alternative alleles, individual i has at QTL q.

H_{iq}: Indicator for heterozygosity. That is, $H_{iq} \in \{0, 1\}$ equals one, if individual i is heterozygous at QTL q.

F_i: Inbreeding coefficient of individual i.

$\overline{f}_i$: Average kinship of individual i with potential mating partners.

Note that $\overline{f}_i$ could in practice be well approximated by the average kinship of individual i with the population.

3.1 Combining Ability

Definition 15. The true combining ability between individuals i and j is defined as

$$T_{ij} \quad = \quad E(\text{TM}(Y_{\text{Off}(i,j)})),$$

where the random K-vector $Y_{\text{Off}(i,j)}$ denotes the vector with trait measurements of a randomly chosen offspring of individuals i and j.

Theorem 5. Suppose that the total merit function is distance-based and piecewise linear, and that all traits are normally distributed. Then, the true combining ability between individuals i and j is

$$T_{ij} = \sum_{o=1}^{O} w_o \mathrm{PT}_{ijo},$$

where the partial combining ability of individuals i and j with respect to breeding objective o equals

$$\mathrm{PT}_{ijo} = \tau_{\max} - \sum_{k=1}^{K} \omega_{ok}\, \psi(\mu_{ijk} - \mathrm{Opt}_{ok}, \sigma_{ijk}).$$

For an additive genetic model,

$$\mu_{ijk} = \mu_k + \frac{\mathrm{TBV}_{ik} + \mathrm{TBV}_{jk}}{2},$$

is the mean, and

$$\sigma^2_{ijk} = \mathrm{MV}_{ik} + \mathrm{MV}_{jk} + \sigma^2_{Ek}$$

is the variance of the trait value of a randomly chosen offspring, σ^2_{Ek} is the environmental variance of trait k, μ_k is the mean of trait k in the base population, and MV_{ik} is the variance of the Mendelian sampling term for trait k that is submitted by animal i to its offspring.

Proof:

Let $Y_{\mathrm{Off}(i,j)}$ denote the random vector with trait measurements of a randomly chosen offspring of individuals i and j. Then,

$$\begin{aligned} T_{ij} &= E(\mathrm{TM}(Y_{\mathrm{Off}(i,j)})) \\ &\overset{\text{Def.11}}{=} E\left(\sum_{o\in\mathcal{O}} w_o \mathrm{PM}_o(Y_{\mathrm{Off}(i,j)})\right) \\ &= \sum_{o\in\mathcal{O}} w_o E(\mathrm{PM}_o(Y_{\mathrm{Off}(i,j)})) \\ &= \sum_{o\in\mathcal{O}} w_o \mathrm{PT}_{ijo}, \end{aligned}$$

where

$$
\begin{aligned}
\mathrm{PT}_{ijo} &= E\left(\mathrm{PM}_o(Y_{\mathrm{Off}(i,j)})\right) \\
&\overset{\text{Def.12}}{=} E\left(\tau_{\max} - \sum_{k=1}^{K} \omega_{ok}\, |Y_{\mathrm{Off}(i,j)k} - \mathrm{Opt}_{ok}|\right) \\
&= \tau_{\max} - \sum_{k=1}^{K} \omega_{ok}\, E(|Y_{\mathrm{Off}(i,j)k} - \mathrm{Opt}_{ok}|) \\
&= \tau_{\max} - \sum_{k=1}^{K} \omega_{ok}\, \psi(\mu_{ijk} - \mathrm{Opt}_{ok}, \sigma_{ijk})
\end{aligned}
$$

where $\mu_{ijk} - \mathrm{Opt}_{ok}$ is the mean and σ^2_{ijk} is the variance of the normally distributed random variable $\Delta_{ijok} = Y_{\mathrm{Off}(i,j)k} - \mathrm{Opt}_{ok}$. The mean and variance can be calculated as follows. The vector $Y_{\mathrm{Off}(i,j)}$ has the representation

$$
Y_{\mathrm{Off}(i,j)k} = \mu_k + \frac{\mathrm{TBV}_{ik} + \mathrm{TBV}_{jk}}{2} + \mathrm{MT}_{ik} + \mathrm{MT}_{jk} + E_k,
$$

where E_k is the environmental effect on the phenotype of the offspring. Consequently, the random variable Δ_{ijok} has mean

$$
\mu_{ijk} - \mathrm{Opt}_{ok} = \mu_k + \frac{\mathrm{TBV}_{ik} + \mathrm{TBV}_{jk}}{2} - \mathrm{Opt}_{ok},
$$

and variance

$$
\sigma^2_{ijk} = \mathrm{MV}_{ik} + \mathrm{MV}_{jk} + \sigma^2_{Ek}.
$$

□

3.2 Simplistic Genetic Model

Lemma 8. Suppose that the total merit function is distance-based and piecewise linear, the traits are normally distributed, and

1) Phenotypic and genetic variances and covariances are constants,

2) The trait optima are several phenotypic standard deviations away from the trait means,

3) The same selection index will be used in all future generations until ζ_n approaches 0.

Then, the partial aggregate genotype for breeding objective o is

$$\mathrm{PT}_{oi} \;=\; v_o(\tilde{\theta}_1)^\top u_i \;=\; \sum_{k=1}^{K} v_{ok}\mathrm{TBV}_{ik},$$

where $v_{ok} = \pm\omega_{ok}$.

Proof:

Assumption 1) implies that $\frac{\partial\sigma^2_{\mathrm{P}nk}}{\partial c_i}(\tilde{c}) = 0$. Consequently, all information on the selection candidates that can affect their aggregate genotypes is given by and $\frac{\partial\mu_{nk}}{\partial c_i}(\tilde{c})$. As the same selection index will be used in all relevant future generations, we have

$$\begin{aligned}\mu_n &= \mu_1 + (n-1)\Delta\mu \\ &= \mu_0 + \mathrm{TBV}^\top c + (n-1)\Delta\mu\end{aligned}$$

where the change $\Delta\mu$ of the population mean from one generation to the next is a constant. Consequently,

$$\frac{\partial\mu_{nk}}{\partial c_i}(\tilde{c}) \;=\; \mathrm{TBV}_{ik}$$

Hence, $u_i = \mathrm{TBV}_i$ is the K-vector with true breeding values of the individual. The corresponding population parameter θ_n with $\frac{\partial\theta_1}{\partial c_i} = \mathrm{TBV}_i$ is $\theta_n = \mu_n$. That is, the state of a population is completely described by the vector with trait means. As the trait optima are several phenotypic standard deviations away from the

trait means, we have approximately $\chi^k_{\mathrm{m}}(\tilde{\xi}_n) = \pm 1$ and $\chi^k_{\mathrm{v}}(\tilde{\xi}_n) = 0$. Moreover,

$$\begin{aligned}
\frac{\partial \mu_{nk}}{\partial \theta_1}(\tilde{\theta}_1) &= \frac{\partial \mu_{nk}}{\partial \mu_1}(\tilde{\mu}_1) \\
&= \left(\frac{\partial \mu_{nk}}{\partial \mu_{11}}(\tilde{\mu}_1), ..., \frac{\partial \mu_{nk}}{\partial \mu_{1K}}(\tilde{\mu}_1) \right) \\
&\overset{(*)}{=} \left(\frac{\partial \mu_{1k}}{\partial \mu_{11}}(\tilde{\mu}_1), ..., \frac{\partial \mu_{1k}}{\partial \mu_{1K}}(\tilde{\mu}_1) \right) \\
&= e_k^\top,
\end{aligned}$$

where $(*)$ holds because μ_n and μ_1 differ only by a constant. Consequently,

$$\begin{aligned}
v_k^{\mathrm{m}}(\tilde{\theta}_1)^\top &\overset{\text{Theo.4}}{=} \sum_{n=1}^{\infty} \zeta_n \, \chi^k_{\mathrm{m}}(\tilde{\xi}_n) \, \frac{\partial \mu_{nk}}{\partial \theta_1}(\tilde{\theta}_1) \\
&= \sum_{n=1}^{\infty} \zeta_n \, (\pm 1) \, e_k^\top \\
&= (\pm 1) \, e_k^\top \sum_{n=1}^{\infty} \zeta_n \\
&= \pm \, e_k^\top
\end{aligned}$$

and

$$\begin{aligned}
v_k^{\mathrm{v}}(\tilde{\theta}_1)^\top &\overset{\text{Theo.4}}{=} \sum_{n=1}^{\infty} \zeta_n \, \chi^k_{\mathrm{v}}(\tilde{\xi}_n) \frac{\partial \sigma_{\mathrm{P}nk}}{\partial \theta_1}(\tilde{\theta}_1) \\
&= \sum_{n=1}^{\infty} \zeta_n \cdot 0 \cdot \frac{\partial \sigma_{\mathrm{P}nk}}{\partial \theta_1}(\tilde{\theta}_1) \\
&= 0^\top.
\end{aligned}$$

The vector with economic weights is thus

$$\begin{aligned}
v_o(\tilde{\theta}_1) &\overset{\text{Theo.4}}{=} \sum_{k=1}^{K} \omega_{ok} \left(v_k^{\mathrm{m}}(\tilde{\theta}_1) + v_k^{\mathrm{v}}(\tilde{\theta}_1) \right) \\
&= \sum_{k=1}^{K} \omega_{ok} \, (\pm \, e_k + 0) \\
&= \sum_{k=1}^{K} \pm \omega_{ok} e_k \\
&= \sum_{k=1}^{K} v_{ok} e_k
\end{aligned}$$

It follows that

$$
\begin{aligned}
\mathrm{PT}_{oi} &\overset{\text{Theo.3}}{=} v_o(\tilde{\theta}_1)^\top u_i \\
&= \left(\sum_{k=1}^{K} v_{ok} e_k^\top\right) \mathrm{TBV}_i \\
&= \sum_{k=1}^{K} v_{ok} \left(e_k^\top \mathrm{TBV}_i\right) \\
&= \sum_{k=1}^{K} v_{ok} \mathrm{TBV}_{ik}
\end{aligned}
$$

□

3.3 General Additive Model

Lemma 9. Let I and J be the parents of a randomly chosen individual from Generation 1. The variance of the breeding values of trait k in Generation 1 equals

$$
\sigma^2_{A1k} = \sigma^2_{\mathrm{PA}1k} + \sigma^2_{\mathrm{MT}1k}
$$

where

$$
\sigma^2_{\mathrm{PA}1k} = \mathrm{Var}\left(\frac{\mathrm{TBV}_{Ik} + \mathrm{TBV}_{Jk}}{2}\right),
$$

is the variance of the parent average, and

$$
\sigma^2_{\mathrm{MT}1k} = \mathrm{Var}\left(\mathrm{MT}_{Ik}\right) + \mathrm{Var}\left(\mathrm{MT}_{Jk}\right)
$$

is the variance of the Mendelian sampling terms that are submitted by the parents to the offspring. In a random mating population,

$$
\sigma^2_{\mathrm{PA}1k} = \frac{1}{4}\mathrm{Var}\left(\mathrm{TBV}_{Ik}\right) + \frac{1}{4}\mathrm{Var}\left(\mathrm{TBV}_{Jk}\right).
$$

Proof:

The breeding value of offspring O has a representation

$$
\mathrm{TBV}_{Ok} = \frac{\mathrm{TBV}_{Ik} + \mathrm{TBV}_{Jk}}{2} + \mathrm{MT}_{Ik} + \mathrm{MT}_{Jk}.
$$

The random variables $\frac{\mathrm{TBV}_{Ik}+\mathrm{TBV}_{Jk}}{2}$, MT_{Ik}, and MT_{Jk} are independent, so

$$\begin{aligned}
\sigma^2_{A1k} &= \mathrm{Var}\left(\mathrm{TBV}_{Ok}\right) \\
&= \mathrm{Var}\left(\frac{\mathrm{TBV}_{Ik}+\mathrm{TBV}_{Jk}}{2} + \mathrm{MT}_{Ik} + \mathrm{MT}_{Jk}\right) \\
&= \mathrm{Var}\left(\frac{\mathrm{TBV}_{Ik}+\mathrm{TBV}_{Jk}}{2}\right) + \mathrm{Var}\left(\mathrm{MT}_{Ik}\right) + \mathrm{Var}\left(\mathrm{MT}_{Jk}\right) \\
&= \sigma^2_{\mathrm{PA1}k} + \sigma^2_{\mathrm{MT1}k}
\end{aligned}$$

The assumption of random mating ensures that $\mathrm{Cov}(\mathrm{TBV}_{Ik}, \mathrm{TBV}_{Jk}) = 0$, so

$$\begin{aligned}
\sigma^2_{\mathrm{PA1}k} &= \mathrm{Var}\left(\frac{\mathrm{TBV}_{Ik}+\mathrm{TBV}_{Jk}}{2}\right) \\
&= \frac{1}{4}\mathrm{Var}\left(\mathrm{TBV}_{Ik}\right) + \frac{1}{4}\mathrm{Var}\left(\mathrm{TBV}_{Jk}\right).
\end{aligned}$$

□

Theorem 6. Suppose that the population is random mating, and that the environmental variance is constant. Let μ_{1k} denote the mean, and $\sigma^2_{\mathrm{P1}k}$ the phenotypic variance of trait k in Generation 1. Let $\sigma^2_{\mathrm{PA1}k}$ and $\sigma^2_{\mathrm{MT1}k}$ be as in Lemma 9 . Then,

$$\begin{aligned}
\frac{\partial \mu_{1k}}{\partial c_i}(\tilde{c}) &= \mathrm{TBV}_{ik}, \\
\frac{\partial \sigma^2_{\mathrm{P1}k}}{\partial c_i}(\tilde{c}) &= \frac{\partial \sigma^2_{\mathrm{PA1}k}}{\partial c_i}(\tilde{c}) + \frac{\partial \sigma^2_{\mathrm{MT1}k}}{\partial c_i}(\tilde{c})
\end{aligned}$$

with

$$\begin{aligned}
\frac{\partial \sigma^2_{\mathrm{PA1}k}}{\partial c_i}(\tilde{c}) &= \frac{1}{2}\left(\left(\mathrm{TBV}_{ik} - 2\tilde{c}_{\mathrm{s}_i}^\top \mathrm{TBV}_k\right)^2 - \left(2\tilde{c}_{\mathrm{s}_i}^\top \mathrm{TBV}_k\right)^2\right), \\
\frac{\partial \sigma^2_{\mathrm{MT1k}}}{\partial c_i}(\tilde{c}) &= 2\mathrm{MV}_{ik}.
\end{aligned}$$

Proof:

$$\mu_{1k}(c) \quad = \quad \mu_{0k} + \sum_i c_i \mathrm{TBV}_{ik},$$

so

$$\frac{\partial \mu_{1k}}{\partial c_i}(\tilde{c}) \quad = \quad \mathrm{TBV}_{ik}.$$

Moreover,

$$\begin{aligned} \sigma^2_{\mathrm{P}1k} &= \sigma^2_{\mathrm{A}1k} + \sigma^2_{\mathrm{E}k}, \\ &\overset{\mathrm{Lem.9}}{=} \sigma^2_{\mathrm{PA}1k} + \sigma^2_{\mathrm{MT}1k} + \sigma^2_{\mathrm{E}k}, \end{aligned}$$

so

$$\frac{\partial \sigma^2_{\mathrm{P}1k}}{\partial c_i} \quad = \quad \frac{\partial \sigma^2_{\mathrm{PA}1k}}{\partial c_i} + \frac{\partial \sigma^2_{\mathrm{MT}1k}}{\partial c_i}$$

with

$$\begin{aligned} \sigma^2_{\mathrm{PA}1k} &\overset{\mathrm{Lem.9}}{=} \frac{1}{4}\mathrm{Var}\left(\mathrm{TBV}_{Ik}\right) + \frac{1}{4}\mathrm{Var}\left(\mathrm{TBV}_{Jk}\right) \\ \sigma^2_{\mathrm{MT}1k} &\overset{\mathrm{Lem.9}}{=} \mathrm{Var}\left(\mathrm{MT}_{Ik}\right) + \mathrm{Var}\left(\mathrm{MT}_{Jk}\right). \end{aligned}$$

Let animal I be of the same sex as animal i. Thus,

$$\begin{aligned} \frac{\partial}{\partial c_i}\sigma^2_{\mathrm{PA}1k} &= \frac{1}{4}\frac{\partial}{\partial c_i}\mathrm{Var}\left(\mathrm{TBV}_{Ik}\right) \\ \frac{\partial}{\partial c_i}\sigma^2_{\mathrm{MT}1k} &= \frac{\partial}{\partial c_i}\mathrm{Var}\left(\mathrm{MT}_{Ik}\right). \end{aligned}$$

Let I_{m} be the set of individuals that have the same sex as individuals i and I. Since

$$\mathrm{Var}\left(\mathrm{TBV}_{Ik}\right) \quad = \quad \sum_{j \in I_{\mathrm{m}}} 2c_j \mathrm{TBV}^2_{jk} - \left(\sum_{j \in I_{\mathrm{m}}} 2c_j \mathrm{TBV}_{jk}\right)^2,$$

we have

$$\begin{aligned}
& \frac{\partial}{\partial c_i}\mathrm{Var}\left(\mathrm{TBV}_{Ik}\right) \\
= \;& 2\mathrm{TBV}_{ik}^2 - 2\left(\sum_{j\in I_\mathrm{m}} 2c_j\mathrm{TBV}_{jk}\right)2\mathrm{TBV}_{ik} \\
= \;& 2\left(\mathrm{TBV}_{ik}^2 - 2\mathrm{TBV}_{ik}\left(\sum_{j\in I_\mathrm{m}} 2c_j\mathrm{TBV}_{jk}\right) + \left(\sum_{j\in I_\mathrm{m}} 2c_j\mathrm{TBV}_{jk}\right)^2 - \left(\sum_{j\in I_\mathrm{m}} 2c_j\mathrm{TBV}_{jk}\right)^2\right) \\
= \;& 2\left(\left(\mathrm{TBV}_{ik} - \left(\sum_{j\in I_\mathrm{m}} 2c_j\mathrm{TBV}_{jk}\right)\right)^2 - \left(\sum_{j\in I_\mathrm{m}} 2c_j\mathrm{TBV}_{jk}\right)^2\right) \\
= \;& 2\left(\left(\mathrm{TBV}_{ik} - 2c_{\mathrm{s}_i}^\top\mathrm{TBV}_k\right)^2 - \left(2c_{\mathrm{s}_i}^\top\mathrm{TBV}_k\right)^2\right)
\end{aligned}$$

and

$$\begin{aligned}
\mathrm{Var}\left(\mathrm{MT}_{Ik}\right) &= \mathrm{E}\left(\mathrm{Var}\left(\mathrm{MT}_{Ik}|I\right)\right) + \mathrm{Var}\left(\mathrm{E}\left(\mathrm{MT}_{Ik}|I\right)\right) \\
&= \mathrm{E}\left(\mathrm{Var}\left(\mathrm{MT}_{Ik}|I\right)\right) + \mathrm{Var}\left(0\right) \\
&= \sum_{i\in I_\mathrm{m}} 2c_i\mathrm{Var}\left(\mathrm{MT}_{ik}\right) \\
&= \sum_{i\in I_\mathrm{m}} 2c_i\mathrm{MV}_{ik}.
\end{aligned}$$

Thus,

$$\frac{\partial}{\partial c_i}\mathrm{Var}\left(\mathrm{MT}_{Ik}\right) = 2\mathrm{MV}_{ik}$$

□

Theorem 7. Let $\sigma^2_{\mathrm{MT}2k}$ be the variance of the Mendelian sampling terms that are received by a randomly chosen individual from generation 2. Suppose that animal i has the same genetic contribution to generation 2 as to generation 1, and that the genetic contribution of an animal is independent from its Mendelian sampling variance. Then,

$$\frac{\partial\sigma^2_{\mathrm{MT}2k}}{\partial c_i} = 2E(\mathrm{MV}_{o_ik}).$$

Proof:

The equation is shown for the case that individual i is a male. The genetic contributions of the animals to Generation 1 are fixed parameters, so for the ease of notation, the animals from Generation 1 can assumed to be ordered such that the set O_i of offspring of animal i is a fixed set. The mating partners are assigned at random. Let I_2 and J_2 be the sire and dam of a randomly chosen individual from Generation 2, and let G denote the genomes of the individuals from Generation 2. We have

$$\begin{aligned}\sigma^2_{\mathrm{MT2}k} &= E\left(\mathrm{Var}\left(\mathrm{MT}_{I_2k}|G\right) + \mathrm{Var}\left(\mathrm{MT}_{J_2k}|G\right)\right)\\ &= E\left(\sum_{l\in I_{1\mathrm{m}}} 2c_l\mathrm{MV}_{lk} + \sum_{l\in I_{1\mathrm{f}}} 2c_l\mathrm{MV}_{lk}\right)\\ &= E\left(2\sum_{l\in I_1} c_l\mathrm{MV}_{lk}\right),\end{aligned}$$

where I_1 is the set of individuals, $I_{1\mathrm{m}}$ is the set of males, and $I_{1\mathrm{f}}$ is the set of females from Generation 1. It is assumed that

$$\sum_{l\in O_i} c_l = c_i + c_{\mathrm{mp}_i},$$

where $c_{\mathrm{mp}_i} = c_i$ is the genetic contribution that is attributed to the mating partners. Thus,

$$\begin{aligned}\sigma^2_{\mathrm{MT2}k} &= 2\sum_{l\in I_1} E(c_l\mathrm{MV}_{lk})\\ &= 2\sum_{i\in I_{0\mathrm{m}}}\sum_{l\in O_i} E(c_l\mathrm{MV}_{lk})\\ &= 2\sum_{i\in I_{0\mathrm{m}}}\sum_{l\in O_i} E(c_l)E(\mathrm{MV}_{lk})\\ &= 2\sum_{i\in I_{0\mathrm{m}}}\sum_{l\in O_i} E(c_l)E(\mathrm{MV}_{o_ik})\\ &= 2\sum_{i\in I_{0\mathrm{m}}} E\left(\sum_{l\in I_2} c_l\right)E(\mathrm{MV}_{o_ik})\\ &= \sum_{i\in I_{0\mathrm{m}}} 2(c_i + c_{\mathrm{mp}_i})E(\mathrm{MV}_{o_ik}).\end{aligned}$$

Consequently,

$$\frac{\partial\sigma^2_{\mathrm{MT2}k}}{\partial c_i} = 2E(\mathrm{MV}_{o_ik}).$$

□

3.4 QTL-based Additive Model with Polygenic Term

Definition 16. The *QTL-based additive model with a polygenic term* assumes that the breeding values of all individuals have the representation

$$\mathrm{TBV}_{ik} = \mathrm{TBV}'_{ik} + \sum_{q=1}^{Q}(X_{iq} - 2p_{0q})a_{qk},$$

where TBV'_{ik} is called the polygenic breeding value, a_{qk} is the true additive effect of QTL q on trait k, and p_{0q} is the frequency of the alternative allele of QTL q in the current generation. Furthermore, the Mendelian sampling term that is transmitted by individual i has the representation

$$\mathrm{MT}_{ik} = \widetilde{\mathrm{MT}}_{ik} + \sum_{q=1}^{Q}\left(U_{iq} - \frac{X_{iq}}{2}\right)a_{qk},$$

where it is assumed that the random allele content $U_{iq} \in \{0,1\}$ of QTL q for the haplotype that was transmitted by individual i, and the part $\widetilde{\mathrm{MT}}_{ik}$ of the Mendelian sampling term that is due to unknown QTLs satisfy the following conditions:

- The random variables U_{iq} and $\widetilde{\mathrm{MT}}_{ik}$ are independent,
- U_{iq} is a Bernoulli variable with $P(U_{iq} = 1) = \frac{X_{iq}}{2}$,
- $\widetilde{\mathrm{MT}}_{ik}$ is a random variable with mean 0 and variance $\frac{1-F_i}{4}\tilde{\sigma}^2_{Ak}$,

where $\tilde{\sigma}^2_{Ak}$ is the polygenic variance of trait k in a non-inbred, unselected and random-mating population.

Theorem 8. For the QTL-based additive model with a polygenic term, the Mendelian sampling variance MV_{ik} of individual i for trait k is

$$\mathrm{MV}_{ik} = \mathrm{Var}\left(\mathrm{MT}_{ik}\right) = \frac{1-F_i}{4}\tilde{\sigma}^2_{Ak} + \sum_{q=1}^{Q}\frac{H_{iq}}{4}a^2_{qk}.$$

Proof:

The Mendelian sampling term of individual i for trait k has the representation

$$\mathrm{MT}_{ik} = \widetilde{\mathrm{MT}}_{ik} + \sum_{q=1}^{Q} Z_{iq} a_{qk},$$

where $Z_{iq} = U_{iq} - \frac{X_{iq}}{2}$. We have

$$\begin{aligned} \mathrm{Var}(Z_{iq}) &= \mathrm{Var}(U_{iq}) = \frac{X_{iq}}{2}\left(1 - \frac{X_{iq}}{2}\right) \\ &= \frac{X_{iq}(2 - X_{iq})}{4} = \frac{H_{iq}}{4}. \end{aligned}$$

The independence of the random variables Z_{iq} and $\widetilde{\mathrm{MT}}_{ik}$ implies that

$$\begin{aligned} \mathrm{Var}\left(\mathrm{MT}_{ik}\right) &= \mathrm{Var}\left(\widetilde{\mathrm{MT}}_{ik}\right) + \sum_{q=1}^{Q} \mathrm{Var}\left(Z_{iq}\right) a_{qk}^2 \\ &= \frac{1 - F_i}{4}\tilde{\sigma}_{Ak}^2 + \sum_{q=1}^{Q} \frac{H_{iq}}{4} a_{qk}^2. \end{aligned}$$

□

Theorem 9. For the QTL-based additive model with a polygenic term, the expected Mendelian sampling variance of an offspring o_i of individual i is

$$E(\mathrm{MV}_{o_i k}) = \frac{1 - \overline{f}_i}{4}\tilde{\sigma}_{Ak}^2 + \sum_{q=1}^{Q} \frac{E(H_{o_i q})}{4} a_{qk}^2,$$

where the expected heterozygosity of an offspring of individual i at QTL q is

$$E(H_{o_i q}) = \frac{X_{iq}}{2}\left(1 - p_{1q}^{\bar{s}_i}(c)\right) + p_{1q}^{\bar{s}_i}(c)\left(1 - \frac{X_{iq}}{2}\right).$$

Thereby, $p_{1q}^{\bar{s}_i}(c)$ is the frequency of the alternative allele of QTL q in the haplotypes from the next generation that are received from parents of the opposite sex.

Proof:

$$\begin{aligned} E(\mathrm{MV}_{o_i k}) &= E\left(\frac{1 - F_{o_i}}{4}\tilde{\sigma}_{Ak}^2 + \sum_{q=1}^{Q} \frac{H_{o_i q}}{4} a_{qk}^2\right) \\ &= \frac{1 - E\left(F_{o_i}\right)}{4}\tilde{\sigma}_{Ak}^2 + \sum_{q=1}^{Q} \frac{E(H_{o_i q})}{4} a_{qk}^2. \end{aligned}$$

The inbreeding coefficient of an individual is the kinship of their parents, so

$$\begin{aligned} E\left(F_{o_i}\right) &= E(f_{iJ}) \\ &= \sum_{j\in Op} 2c_j f_{ij} \\ &= \overline{f}_i, \end{aligned}$$

where J is a randomly chosen mating partner, and Op is the set of individuals with the opposite sex as individual i. Furthermore, the probability that the offspring is heterozygous at QTL q is

$$\begin{aligned} E(H_{o_iq}) &= P(H_{o_iq}=1) \\ &= \frac{X_{iq}}{2}\Big(1-p_{1q}^{\bar{s}_i}(c)\Big)+p_{1q}^{\bar{s}_i}(c)\left(1-\frac{X_{iq}}{2}\right), \end{aligned}$$

where the first summand is the probability that individual i submits allele 1, and the mating partner submits allele 0, and the second summand is the probability that individual i submits allele 0, and the mating partner submits allele 1.

□

Theorem 10. For a vector $c \in \mathcal{C}$ with genetic contributions of the selection candidates let $\overline{f}_i(c)$ be the average kinship of individual i with potential mating partners, let

$$\overline{F}_1(c) \quad = \quad \sum_i c_i \overline{f}_i(c),$$

be the average inbreeding coefficient in the next generation, let

$$\overline{F}_1^P(c) \quad = \quad \sum_i c_i F_i,$$

be the average inbreeding coefficient in the parents of the next generation, let

$$p_{1q}(c) \quad = \quad \sum_i c_i \frac{X_{iq}}{2}$$

be the expected frequency of QTL q in the next generation, and let

$$\overline{H}_{1q}^P(c) = \sum_i c_i H_{iq}$$

be the average heterozygosity of QTL q in the parents of the next generation. Furthermore, let $\tilde{c} \in \mathcal{C}$ be the vector with default contributions of the individuals. Then,

$$\begin{aligned}
\frac{\partial \overline{F}_1}{\partial c_i}(\tilde{c}) &= 2\overline{f}_i(\tilde{c}), \\
\frac{\partial \overline{F}_1^P}{\partial c_i}(\tilde{c}) &= F_i, \\
\frac{\partial p_{1q}}{\partial c_i}(\tilde{c}) &= \frac{X_{iq}}{2}, \\
\frac{\partial \overline{H}_{1q}^P}{\partial c_i}(\tilde{c}) &= H_{iq}.
\end{aligned}$$

Proof:

Only the first equation needs a proof. We have

$$\overline{f}_l(c) \quad = \quad \sum_{j \in Op_l} 2 c_j f_{lj}$$

where Op_l is the set of individuals with the opposite sex as individual l. Thus,

$$
\begin{aligned}
\overline{F}_1(c) &= \sum_l c_l \sum_{j \in Op_l} 2c_j f_{lj} \\
&= \sum_{l \in M} c_l \sum_{j \in F} c_j 2 f_{lj} + \sum_{l \in F} c_l \sum_{j \in M} c_j 2 f_{lj} \\
&= \sum_{l \in M} \sum_{j \in F} c_l c_j 2 f_{lj} + \sum_{l \in F} \sum_{j \in M} c_l c_j 2 f_{lj} \\
&= \sum_{j \in M} \sum_{l \in F} c_j c_l 2 f_{jl} + \sum_{l \in F} \sum_{j \in M} c_l c_j 2 f_{lj} \\
&= \sum_{j \in M} \sum_{l \in F} 2c_j 2c_l f_{jl},
\end{aligned}
$$

where M is the set of males, and F is the set of females. As the inbreeding coefficient of an individual equals the kinship of the parents, this equation shows that $\overline{F}_1(c)$ is indeed the average inbreeding coefficient in the next generation. Furthermore, it follows that

$$
\begin{aligned}
\overline{F}_1(c) &= \sum_{j \in M} 2c_j \sum_{l \in F} 2c_l f_{jl} \\
&= \sum_{j \in M} 2c_j \overline{f}_j(c),
\end{aligned}
$$

Consequently, if individual i is a male, then $\overline{f}_j(c)$ does not depend on c_i, so

$$
\frac{\partial \overline{F}_1}{\partial c_i}(\tilde{c}) = 2\overline{f}_i(\tilde{c}),
$$

The proof for females is analogous.

□
